# Allatostatin A Signalling in *Drosophila* Regulates Feeding and Sleep and Is Modulated by PDF

**DOI:** 10.1371/journal.pgen.1006346

**Published:** 2016-09-30

**Authors:** Jiangtian Chen, Wencke Reiher, Christiane Hermann-Luibl, Azza Sellami, Paola Cognigni, Shu Kondo, Charlotte Helfrich-Förster, Jan A. Veenstra, Christian Wegener

**Affiliations:** 1 Neurobiology and Genetics, Theodor-Boveri-Institute, Biocenter, University of Würzburg, Würzburg, Germany; 2 INCIA, UMR 5287 CNRS, University of Bordeaux, Talence, France; 3 Department of Zoology, University of Cambridge, Cambridge, United Kingdom; 4 Genetic Strains Research Center, National Institute of Genetics, Shizuoka, Japan; Washington University in Saint Louis School of Medicine, UNITED STATES

## Abstract

Feeding and sleep are fundamental behaviours with significant interconnections and cross-modulations. The circadian system and peptidergic signals are important components of this modulation, but still little is known about the mechanisms and networks by which they interact to regulate feeding and sleep. We show that specific thermogenetic activation of peptidergic Allatostatin A (AstA)-expressing PLP neurons and enteroendocrine cells reduces feeding and promotes sleep in the fruit fly *Drosophila*. The effects of AstA cell activation are mediated by AstA peptides with receptors homolog to galanin receptors subserving similar and apparently conserved functions in vertebrates. We further identify the PLP neurons as a downstream target of the neuropeptide pigment-dispersing factor (PDF), an output factor of the circadian clock. PLP neurons are contacted by PDF-expressing clock neurons, and express a functional PDF receptor demonstrated by cAMP imaging. Silencing of AstA signalling and continuous input to AstA cells by tethered PDF changes the sleep/activity ratio in opposite directions but does not affect rhythmicity. Taken together, our results suggest that pleiotropic AstA signalling by a distinct neuronal and enteroendocrine AstA cell subset adapts the fly to a digestive energy-saving state which can be modulated by PDF.

## Author Summary

Feeding and sleep are fundamental behaviours that are controlled by diverse neuropeptides. While feeding is associated with wake periods, sleep prevents feeding. Both feeding and sleep are timed to specific parts of the day by internal clocks, presumably to optimise behaviour and metabolic processes. We investigated the functions of Allatostatin A (AstA) peptides in the fruit fly. AstA is produced by neurons as well as by endocrine cells in the midgut epithelium. Thermogenetic activation of subsets of AstA-producing cells in the brain and midgut revealed that AstA affects both feeding and sleep in opposite directions: feeding is reduced by AstA signalling, while sleep is promoted. Others could previously show that insect AstA also inhibits gut motility and release of digestive enzymes from the gut. An attractive conclusion is that AstA signalling helps to put flies in a digestive energy-saving state. Interestingly, a set of six AstA-expressing PLP neurons are in close contact to neurons central to the circadian clock network, and express functional receptors for the clock output signal PDF. This opens the possibility that PLP neurons are at the interface between clock, feeding and sleep—a hypothesis that needs to be tested in the future.

## Introduction

Neuropeptides and peptide hormones transfer a wide variety of neuronal or physiological information from one cell to the other by activating specific receptors on their target cells [1]. Most if not all peptides are pleiotropic and can orchestrate diverse physiological, neuronal or behavioural processes [2,3]. In vertebrates, such a pleiotropic effect is especially prominent in the regulation of feeding and sleep. Many different peptides (e.g. orexin/hypocretin, ghrelin, obestatin) modulate different aspects of both behaviours [4,5], which reciprocally influence each other [6,7]. The temporal pattern of neuroendocrine activity and neuropeptide release is shaped by sleep homeostasis and the circadian clock which, in turn, reciprocally affects feeding and sleep-wake cycles [7–9]. Significant progress has been made in this field during recent years. Still little characterised, however, is the neuronal architecture that enables the relevant peptidergic neurons to integrate energy status, circadian time and sleep-wake status in order to coordinate the timing of sleep, locomotor activity and feeding. Information about the output signals by which endogenous clocks provide time- and non-circadian information to relevant peptidergic cells is still limited.

During the last years, the fruit fly *Drosophila* has become an important model for research into the regulation of feeding and sleep [10–13]. *Drosophila* offers advanced genetic tools, a small brain with only about 100.000 neurons and a quantifiable sleep- and feeding behaviour that shows characteristics very similar to that of mammals [11,14,15]. These features greatly facilitate the analysis of the neuronal and endocrine underpinnings of feeding and sleep. Like in most animals, feeding and sleep follow a circadian pattern in the fruit fly [16–18] with little characterised neuronal and hormonal pathways downstream of the central clock. Like in mammals, a number of neuropeptides have been shown to be involved in the regulation of feeding [11,12] or sleep [19,20] in *Drosophila*. Yet, so far, only sNPF [21–24] and likely also NPF [25,26] are implicated in the regulation of both feeding and sleep. Also Insulin-like peptide (DILP)-expressing neurons (IPCs) in the pars intercerebralis affect feeding and sleep, yet only feeding seems to be directly dependent on DILP signalling [27].

Recent work by Hergarden and colleagues demonstrated that neurons expressing neuropeptides of the allatostatin A (AstA) family regulate feeding behaviour of the fruit fly [28]. Constitutive activation of AstA cells contained in the *AstA*^*1*^*-Gal4* expression pattern by ectopic expression of the bacterial low threshold voltage-gated NaChBac channel [29] potently inhibited starvation-induced feeding. In contrast, constitutive inactivation of AstA^1^ cells by expression of the inwardly rectifying Kir2.1 potassium channel [30] increased feeding under restricted food availability. NaChBac activation of AstA^1^ cells also inhibited the starvation-induced increase of the proboscis extension reflex (PER), a behavioural indicator for glucose responsiveness [28]. The AstA^1^ expression pattern includes a large number of brain neurons plus gut-innervating thoracico-abdominal ganglion (TAG) neurons and enteroendocrine cells (EECs) in the posterior midgut [28]. This broad expression pattern is consistent with earlier described patterns of AstA-like immunoreactivity [31–34] and suggests multiple functions for AstA. Earlier work had demonstrated an effect of AstA on gut motility [35]. Two AstA receptors, DAR-1 (= AlstR) and DAR-2 are characterised for *Drosophila* [36–39]. Different genome-based phylogenetic GPCR analyses independently demonstrated their homology with the galanin receptor family of vertebrates [40–43]

Using anatomical subdivision and genetic manipulation of neuronal activity, we aimed to identify AstA functions and -if possible- assign them to subsets of AstA expressing cells. Our results revealed new interconnected AstA functions that link feeding and sleep and identify AstA-expressing PLP neurons and EECs as a target of the central clock output factor PDF. Pleiotropic AstA signalling seems capable of coordinating multiple aspects of physiology and behaviour in a coherent manner to adapt the fly to a digestive energy-saving state. The functional range of AstA signalling in the fly is thus reminiscent of the pleiotropy found in mammalian galanin signalling [44–46].

## Results

To be able to restrict genetic manipulations to subgroups of AstA-expressing cells in *Drosophila*, we first generated an *AstA*^*34*^-*Gal4* line that specifically drive ectopic expression of effector genes in restricted subsets of AstA-expressing cells.

### Expression pattern of the AstA^34^-Gal4 line

**Fig 1:**
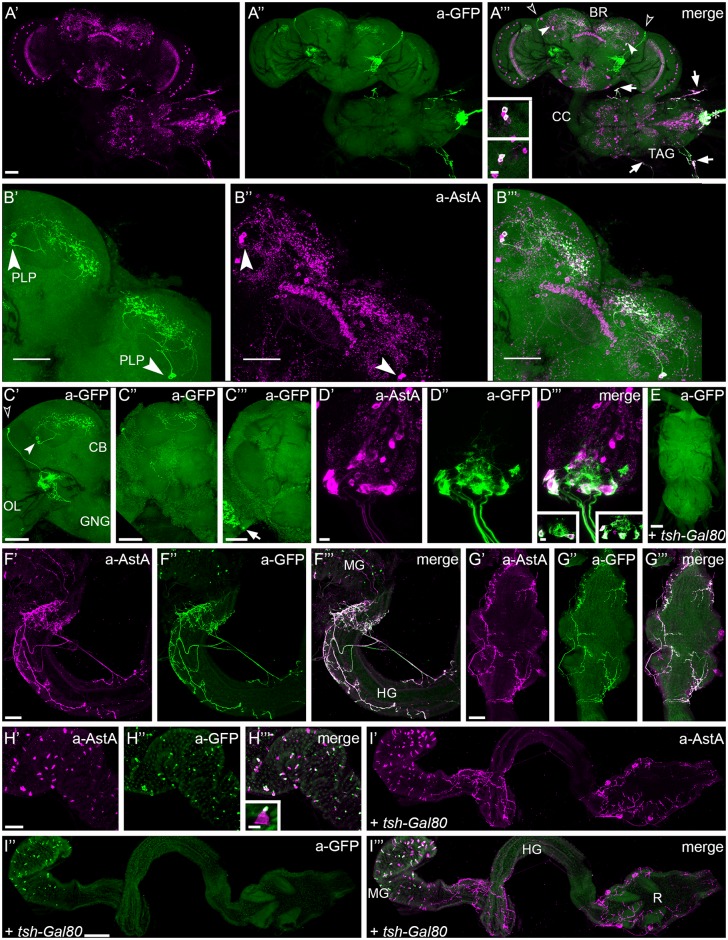
AstA (magenta) and GFP (green) immunolabeling of nervous systems and guts of adult AstA^34^>GFP (A–D, F–H) and tsh-Gal80; AstA^34^>GFP (E, I) flies. **(A)** GFP expression is detectable in two paired groups of brain neurons. In each hemisphere, one group with two somata in the posterior lateral protocerebrum (PLP cells, solid arrowheads in **A'''**) and a second group with two to four somata in the lateral cell body rind (LCBR cells, open arrowheads in **A'''**) are labelled (see also **C**). The LCBR neurons are anti-AstA-negative. Some of the AstA-IR medulla neurons also express GFP. In the abdominal TAG, the six AstA-IR DLAa cells show strong anti-GFP staining and project through the median abdominal nerve towards the gut (asterisk in **A'''**). Four peripheral cells located on nerves that exit the TAG dorso-laterally also exhibit co-labeling (arrows in **A'''**). Inset in **A'''**: Single optical sections showing double-labelled PLP cells of both hemispheres. **(B)** Close-up of the PLP neurons (solid arrowheads in **B',B''**) from **A**, maximum projection of the horizontal sections between the level of central body and calyx. The extensive arborisations in the superior protocerebrum are visible. **(C)** The GFP signal in brains of different individuals illustrates the variability of expression intensity among PLP, LCBR and medulla neurons. The PLP cells (solid arrowhead in **C'**) innervate the superior protocerebrum, while the ramifications of the LCBR neurons (open arrowhead in **C'**) lie mainly within the posterior lateral protocerebrum and the posterior slope. **C'** and **C''** show a strong and moderate GFP-expression intensity, respectively. The arrow in C''' marks a strongly stained neuron in the medulla, which occurred only in a few preparations **(D)** Detail of the abdominal TAG. Three pairs of AstA-IR DLAa neurons co-express GFP and run through the median abdominal nerve to innervate the hindgut and posterior midgut (see **F**). The membrane-targeted GFP distinctly marks the projections of these neurons. Two single optical sections (insets in **D'''**) reveal six co-labelled cell bodies. **(E)** AstA34-Gal4 expression in the TAG is absent with *tsh-Gal80*. **(F)** AstA and GFP labeling are present in neuronal processes at the hindgut, which extend onto the posterior midgut (Malpighian tubules have been removed during dissection). **(G)** The rectal part of the hindgut is likewise innervated by double-labelled neurons. **(H)** GFP is expressed in most of the AstA-producing EECs that are scattered within the epithelium of the posterior midgut. The maximum intensity projection of a single EEC in the inset of **H'''** illustrates that the main GFP signal is restricted to the narrow apical portion of the cells. **(I)** GFP expression is absent from gut neurons in individuals carrying *tsh-Gal80*, but remains in the AstA EECs. (Malpighian tubules have been removed during dissection.) Scale bars: in A-C and E–H 50 μm; in D 10 μm; in I 100 μm. BR brain, CB central brain, CC cervical connective, IL ileum, MG midgut, OL optic lobe, PV pyloric valve, R rectum, RV rectal valve, GNG gnathal ganglia, TAG thoracico-abdominal ganglion.

To test the specificity of *AstA*^*34*^*-Gal4* expression in adult flies, we co-immunolabelled AstA^34^>*GFP* flies against GFP and AstA. The observed AstA immunoreactivity (IR) pattern was consistent with earlier descriptions [31–33] (Fig 1), and we adopted the nomenclature of Yoon and Stay (1995). S1 Table provides a summary of the localisation of *AstA*^34^*-Gal4*-driven GFP expression in relation to the AstA IR.

In each brain hemisphere of *AstA*^*34*^>*GFP* flies, GFP was consistently detected in two to three of the three AstA-IR PLP interneurons with somata in the posterior lateral protocerebrum (Fig 1A and 1B). These cells sent a primary neurite dorsally just anterior of the calyx which typically trifurcated and then extensively arborised throughout the whole superior lateral (SLP), superior intermediate (SIP) and superior medial (SMP) protocerebrum (Fig 1A and 1B, S1 and S2 Movies). In the anterior-posterior axis, this large arborisation field extended from the height of the fan-shaped body to just anterior of the calyx. Furthermore, GFP was found in two to four cells per hemisphere with somata in the lateral cell body rind close to the lateral horn. These LCBR neurons were AstA immunonegative and are not contained in the *AstA*^*1*^*-Gal4* line (Fig 1A and 1B). In addition, a varying small number of AstA-IR neurons in the medulla showed generally weak GFP expression (Fig 1A and 1C). In some preparations, single medulla neurons were found that exhibited a stronger GFP signal (Fig 1C).

In the thoracico-abdominal ganglion (TAG), three pairs of AstA-IR DLAa cells within the posterior abdominal region ([27], Fig 1A and 1D) sent neurites via the median abdominal nerve to innervate the hindgut and the posterior-most midgut (Fig 1F, 1G and 1I). Regions with innervations include the pyloric valve and the rectal valve, which control transit of gut contents and urine from the midgut to the ileum and from the ileum to the rectum. Processes of the DLAa neurons innervating the rectum in part extend through the muscle layer (Fig 1G), thus their peptide signals might target the rectal epithelium. The DLAa neurons consistently exhibited strong AstA^34^-driven GFP expression, while the brain neurons showed a more variable GFP labelling intensity between preparations (see Fig 1C). In many preparations, one or a few variably positioned non-AstA-IR interneurons within the TAG additionally showed a weak GFP signal.

Outside of the CNS, two pairs of peripheral AstA-IR neurons with somata located on the segmental nerves leading to the wings and the halteres [33] expressed GFP (Fig 1A). Furthermore, GFP was detectable in most if not all AstA-IR EECs in the posterior part of the midgut (Fig 1F–1H). The staining results are summarized in S1 Table.

In comparison to the *AstA*^*34*^*-Gal4* pattern, the expression pattern of *AstA*^*1*^*-Gal4* included the following AstA-IR neurons per brain hemisphere: all three PLP neurons, 2 neurons in the superior protocerebrum, ~ 30 medulla neurons, and three neurons with cell bodies in the GNG (gnathal (= subesophageal) ganglion) thought to be important for sucrose responsiveness [28]. Thus, *AstA*^*1*^*-Gal4* drives expression in a larger number of AstA brain neurons though it is lacking the AstA-negative LCBR brain neurons (S1 Table). The expression in the TAG is identical in both *AstA-Gal4* lines, while *AstA*^*34*^*-Gal4* includes a larger fraction of AstA EECs in the midgut. A schematic summary of the expression patterns is given in S2 Fig.

### Activation of the AstA PLP neurons and EECs is sufficient to reduce food intake

**Fig 2:**
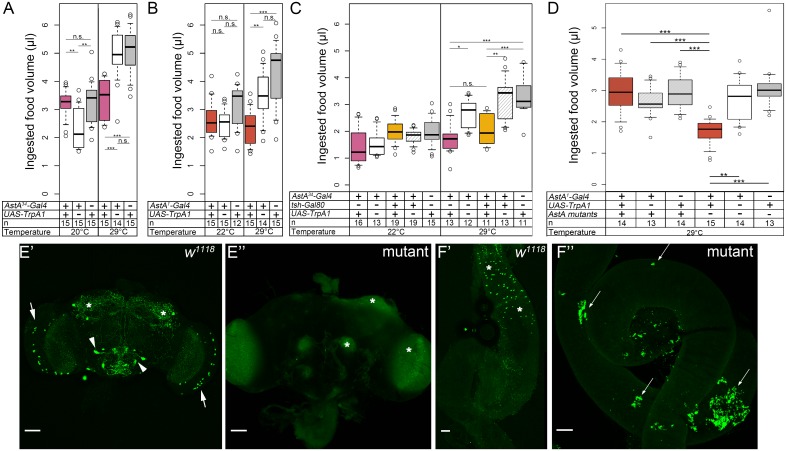
Thermogenetic activation of the AstA cells resulted in reduced food intake. The total food volume consumed within two days was measured via the CAFE assay. At 20°C, *AstA*^*34*^>*TrpA1* **(A)**, *AstA*^*1*^*>TrpA1* **(B)** and *tsh-Gal80; AstA*^*34*^>*TrpA1***(C)** flies did not consume less food than the controls. Activation of the TrpA1 channel at 29°C resulted in significantly reduced food intake in *AstA*^*34*^>*TrpA1* **(A)**, *AstA*^*1*^*>TrpA1* **(B)** flies compared to the controls. Food intake of *tsh-Gal80; AstA*^*34*^>*TrpA1* **(C)** flies was significantly lower than in control flies, but not different from *AstA*^*34*^>*TrpA1* flies. **(D)** Thermogenetical activation of AstA^1^ cells did not reduce food intake in in flies with a AstA^SK4^ null mutant background, in contrast to flies with an *AstA* wildtype background tested in parallel. **(E)** Maximum projections of confocal stacks of an adult brain of a w^1118^ control **(E')** and an AstA^SK4^ mutant **(E'')** immunostained against AstA. **(F)** AstA immunostaining in the midgut of a w^1118^ control **(F')** and AstA^SK4^ mutant **(F'')**. AstA-IR neurons are visible in the optic lobes (arrow), gnathal ganglion (arrow head) and superior protocerebrum (asterisks) in the brain **(E')**, and in EECs in the posterior midgut (asterisks in **F'**). In contrast, AstA -IR cells are absent in the mutant, indicating a global lack of AstA peptides. The mutant tissues were scanned at very high gain to detect even weak potential AstA immunostaining. This led to detection of autofluorescence signals in the brain (asterisks in **E''**) and gut associated fat body (arrows in **F''**). Scale bars: 50 μm.

To test for a possible role of *AstA*^*34*^ cells in the control of food intake, we employed the CAFE assay [47] and measured food intake while AstA^34^ cells were conditionally activated by the thermogenetic effector TrpA1. TrpA1 is a temperature sensor widely used to conditionally activate neurons by temperatures above 28°C [48,49]. Male *AstA*^*34*^>*TrpA1* flies were raised on food at 20 or 22°C, and then assayed over a period of two days. At 29°C, but not at 20°/22°C, food consumption was significantly lowered in *AstA*^*34*^>*TrpA1* flies (Fig 2A). A similar reduction of food intake at 29°C was detected for *AstA*^*1*^>*TrpA1* flies (Fig 2B). This effect is not sex-specific, as a similar significant reduction in food intake was also observed in females (S3 Fig). These results are consistent with a previous report showing reduced starvation-induced feeding upon constitutive activation of AstA cells by *AstA*^*1*^*>NaChBac* in a different feeding assay [28]. These findings indicate that the LCBR neurons (lacking in *AstA*^*1*^) and AstA cells in the GNG (lacking in *AstA*^*34*^) are dispensable to reduce food intake. Thus, activation of only the AstA^34^ subset appears sufficient to reduce food intake.

To restrict the activation pattern further, we created *tsh-Gal80; AstA*^*34*^*>TrpA1* (*UAS-TrpA1/tsh-Gal80; AstA*^*34*^*-Gal4/+*) flies. *tsh-Gal80* suppresses *Gal4* expression in the thoracic and abdominal part of the CNS [50–52], and limited *TrpA1* expression to AstA^34^ central brain neurons and EECs (Fig 1E and 1I). Thermogenetic activation of this AstA^34^ cell subset by a shift to 29°C was sufficient to reproduce the feeding phenotype found in *AstA*^*34*^*>TrpA1* flies (Fig 2C), indicating that the AstA neurons in the TAG and periphery are dispensable for feeding inhibition. A role for the AstA neurons in the optic lobe seems very unlikely due to their anatomy and since *AstA*^*34*^*-Gal4* driven expression in these neurons was inconsistent and weak and comprised only few of the many AstA optic lobe neurons. Thus, we conclude that the AstA-producing PLP cells and/or EECs are sufficient to control food intake.

So far, we had observed feeding inhibition upon activation of AstA cells. Inhibition of AstA^1^ cells by constitutive expression of *UAS-Kir2*.*1* [30] has previously been reported to increase feeding under restricted food availability [28]. To exclude developmental effects due to constitutive silencing, we next conditionally manipulated AstA cells using the TARGET system [53]. At both 18°C and 30°C, *tubGal80*^*ts*^*;AstA*^*34*^*>Kir2*.*1* flies showed a similar food intake as controls under non-restricted food availability in the CAFE assay (S4 Fig). This suggests to us that signalling from PLP neurons or EECs is not essential for normal feeding behaviour and that PLP neurons and EECs are not core components of a feeding circuit. Rather, AstA cells modulate feeding circuits, and likely become functionally active only under specific circumstances, e.g. when flies are satiated or feeding will interfere with other behaviours. A similar situation has been found for *hugin*-expressing neurons in the *Drosophila* larva. When activated via TRPA1, they inhibit fictive pharyngeal pumping. When silenced or ablated, fictive pharyngeal pumping is unchanged compared to controls, suggesting a modulatory role of the anorexigenic *hugin* pyrokinin peptide [54].

### Reduced food intake upon AstA cell activation can be traced to Allatostatin A signalling

Peptides are typically co-localised with other peptides or classic transmitters [55,56]. To identify whether the observed feeding phenotype upon activation of AstA cells is due to AstA or a co-localised peptide/transmitter, we used *AstA*^*SK4*^ null mutant flies generated by germline-specific CRISPR/Cas9 [57]. In contrast to controls, *AstA*^*SK4*^ mutants are devoid of any AstA-IR in the nervous system and gut (Fig 2E and 2F). We then thermogenetically activated the AstA^1^ neurons in an *AstA* null mutant background and found no difference in food uptake compared to controls (Fig 2D). Similar observations were made when reducing *AstA* expression by RNAi in *AstA*^*34*^>*TrpA1/ AstA-RNAi* flies (S5A Fig). Together, these experiments show that PLP neurons or EECs signal via AstA peptides to reduce food intake. The general lack of AstA without activation of AstA cells did, however, not reduce feeding under the experimental conditions; controls in wildtype and AstA^SK4^ mutant background showed similar amounts of ingested food (Fig 2D).

### Activation of AstA PLP neurons and EEC decreases locomotor activity and promotes sleep

**Fig 3:**
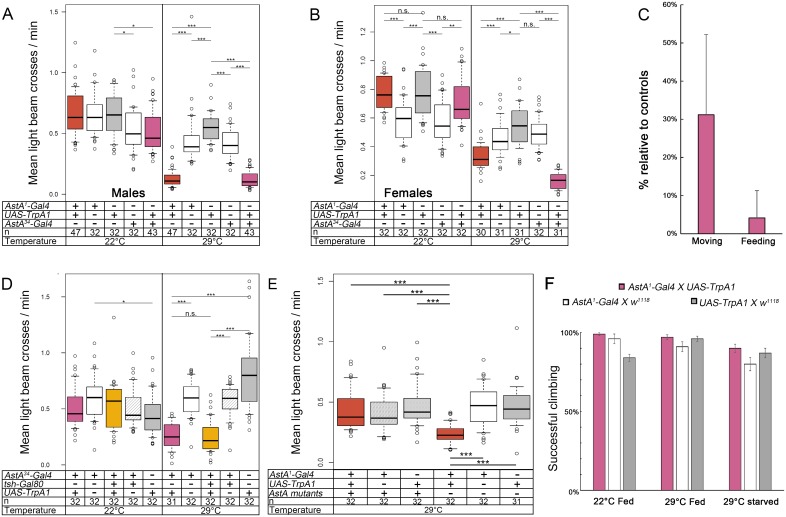
Thermogenetic activation of AstA cells resulted in strongly inhibited locomotor activity. At 29°C, the activity levels of *AstA*^*1*^>*TrpA1* and *AstA*^*34*^>*TrpA1* males **(A)** and females **(B)** were significantly lower than in controls. At 22°C, *AstA*^*1*^>*TrpA1* and *AstA*^*34*^>*TrpA1* flies showed the same activity level than the controls. **(C)** Manual behavioural categorisation of individual flies monitored for 4h by a camera in the CAFE assay (n = 3, see S7 Fig). Both movement and food consumption of *AstA*^*1*^*>TrpA1* flies were strongly reduced relative to *AstA*^*1*^ x *w*^*1118*^ controls at 29°C. n = 3 **(D)** The activity level of *tsh-Gal80; AstA*^*34*^>*TrpA1* flies was similar to *AstA*^*34*^>*TrpA1* males and lower compared to controls. **(E)** Activation of AstA^1^ cells did not reduce locomotory activity in flies with an AstA^SK4^ null background, in contrast to flies with an *AstA* wildtype background tested in parallel. **(F)** In a negative geotaxis assay, starved or satiated *AstA*^*1*^*>TrpA1* flies show a similar locomotor performance compared to control flies at 22°C and 29°C. p ≤ 0.05. ** p ≤ 0.01, *** p ≤ 0.001.

Locomotor activity affects energy expenditure and consequently also appetite, and is in turn altered by hunger and feeding. We therefore asked whether activation of AstA cells affects locomotor activity. Flies were kept in small glass tubes on agar-sucrose food and their locomotor activity was monitored using the DAM system. Compared to controls, the average locomotor activity of *AstA*^*1*^>*TrpA1* and *AstA*^*34*^*>TrpA1* flies was strongly and significantly reduced at 29°C, but not at 22°C in both sexes (Fig 3A and 3B, S6 Fig). In contrast, *AstA*^*1*^*>TrpA1* flies were not impaired in climbing ability in a startle-induced negative geotaxis assay, independent of being fed or starved for 24h at 29°C (Fig 3F), showing that the flies were not suffering from impaired locomotor ability or energy deficiency due to decreased feeding.

To analyse locomotor activity in the CAFE assay, we video-monitored activity of *AstA*^*1*^>*TrpA1* males in a slightly modified setup using Petri dishes instead of a 24 well plate. Prior to testing, flies were starved for 24h at 29°C but had free access to water. After placement into the Petri dish, we filmed pairs of flies at 29°C for 4 hours and visually categorised their behaviour (not moving, moving, feeding). Fig 3C shows that *AstA*^*1*^*>TrpA1* spent much less time moving as well as feeding compared to *AstA*^*1*^ x *w*^*1118*^ controls, with individual variations within both strains (S7 Fig). Nevertheless, *AstA*^*1*^*>TrpA1* flies were fully capable of locating the capillary and did not stay there longer than controls, which would have allowed them to feed without moving (S7 Fig).

**Fig 4:**
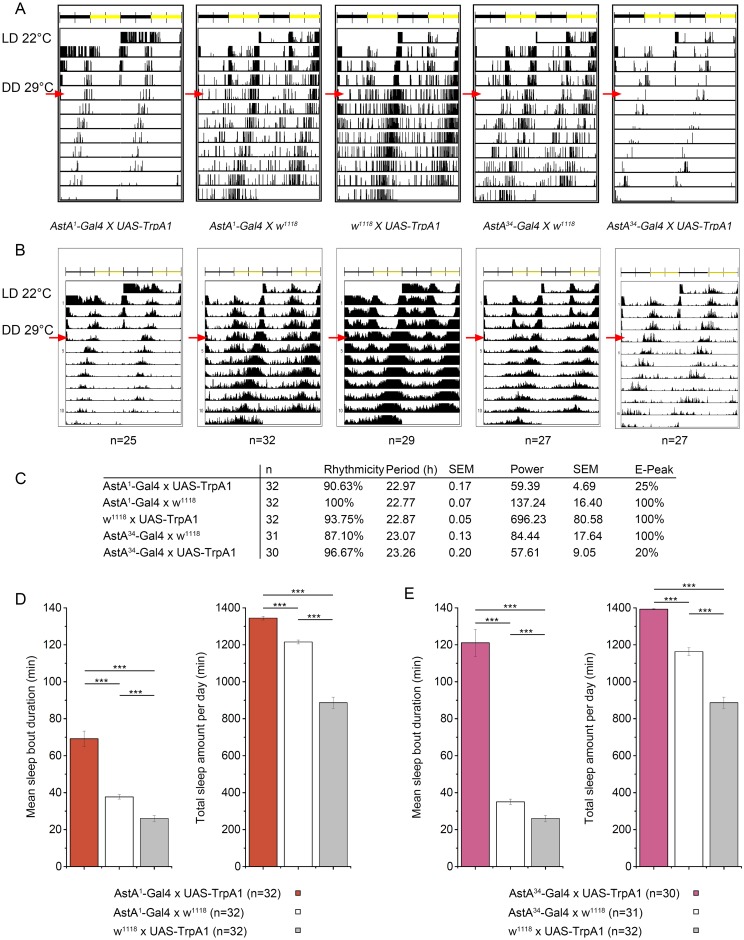
Locomotor activity in the DAM system under constant conditions. **(A)** Typical double-plotted actograms of *AstA*^*34*^>*TrpA1*, *AstA*^*1*^*>TrpA1* and control flies kept for three days at 22°C and L:D 12:12, then switched to 29°C and constant darkness (DD, red arrows). **(B)** Average actograms for all rhythmic flies tested (non-rhythmic flies were excluded). Flies with activated AstA cells showed not only a decreased activity, but also a lack of evening activity. **(C)**The rhythmicity and period is unchanged compared to controls. **(D-E)** Total sleep amount and sleep bound duration is significantly increased upon AstA^1^ **(D)** and Asta^34^ **(E)** cell activation in DD.

We next monitored the locomotor activity of *tsh-Gal80; AstA*^*34*^>*TrpA1* flies (Fig 3D) and found a reduction of locomotor activity similar to *AstA*^*1*^>*TrpA1* flies upon thermogenetic activation. (Fig 3A). Activation of PLP neurons and/or the AstA EECs seems thus sufficient to reduce locomotor activity. The inhibitory effect is again mediated by AstA peptide signalling, since thermogenetic activation of AstA cells in *AstA*^*1*^>*TrpA1* flies in the AstA^SK4^ null mutant background did not significantly alter locomotor activity (Fig 3E). The rhythmicity and period of locomotor activity [58] was not affected by activation of AstA cells in *AstA*^*1*^>*TrpA1* and *AstA*^*34*^>*TrpA1* flies at 29°C and constant darkness (Fig 4). Strikingly, however, subjective evening activity was lost. (Fig 4A and 4B). A general lack of AstA without activation of AstA cells did not influence locomotor activity, as controls in wildtype and AstA^SK4^ mutant background showed similar activity levels (Fig 3E).

**Fig 5:**
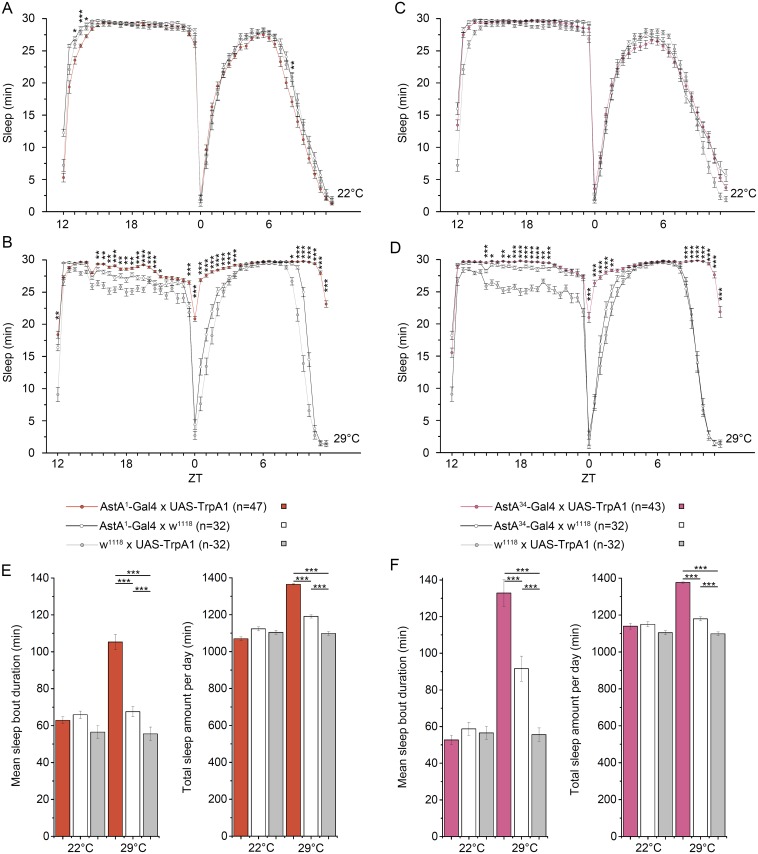
Thermogenetic activation of AstA cells strongly promoted sleep. At 22°C, *AstA*^*1*^>*TrpA1* **(A)** and *AstA*^*34*^>*TrpA1* male flies **(C)** did not sleep more than controls. Activation of the TrpA1 channel at 29°C resulted in increased sleep time of *AstA*^*1*^>*TrpA1* **(B)** and *AstA*^*34*^>*TrpA1* **(D)** flies especially during the time of the morning and evening activity. For both *AstA*^*1*^>*TrpA1* **(E)** and *AstA*^*34*^>*TrpA1* **(F)**, mean sleep bout duration and the total amount of sleep per day was significantly increased when AstA cells were thermogenetically activated.

**Fig 6:**
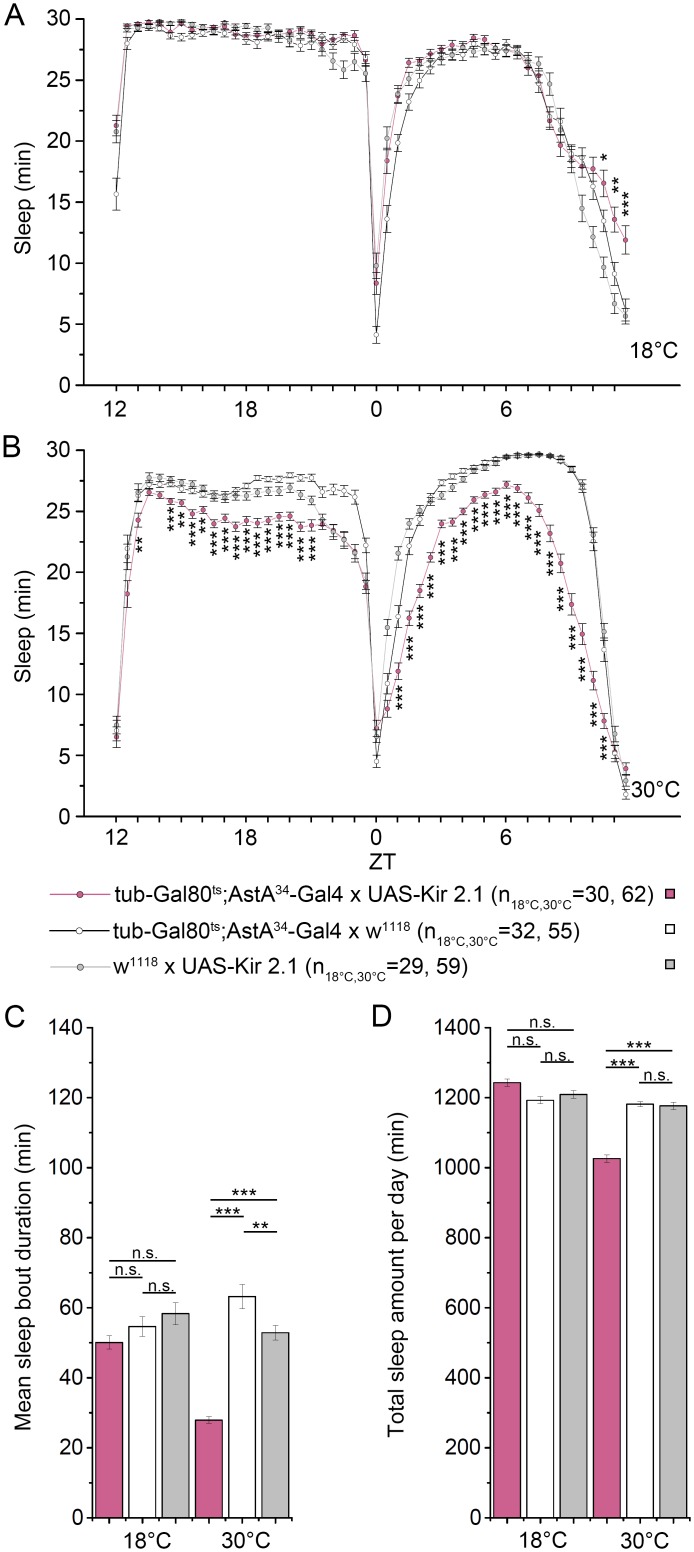
Conditional silencing of AstA^34^ cells by ectopic expression of the inward rectifying K^+^ channel Kir2.1 decreases sleep. **(A+B)** Averaged sleep over 24h of *tubGal80*^*ts*^; *AstA*^*34*^*>Kir2*.*1* experimental flies and controls. **(C+D)** Average sleep bout duration and total amount of sleep calculated from A+B. At 18°C, *tubGal80*^*ts*^ inhibits ectopic expression of Kir2.1 and experimental flies show a similar sleep behaviour as controls **(A, C-D)**. At 30°C, Kir2.1 is expressed in AstA^34^ cells and causes a significant reduction of total sleep and average sleep bout duration **(C+D)**, both during the light and dark phase (**B**). p ≤ 0.05. ** p ≤ 0.01, *** p ≤ 0.001.

A strongly reduced locomotor activity is suggestive of abnormal sleep. Applying the widely used 5 min inactivity criterion [59], we found that in fact thermogenetic activation of the AstA^1^ and AstA^34^ cells strongly promotes sleep, which is most apparent during the morning and evening activity peaks in both males (Fig 5) and females (S8 Fig). At 29°C, but not at 22°C, *AstA*^*1*^*>TrpA1* and *AstA*^*34*^*>TrpA1* flies showed a significant increase in both total amount of sleep and sleep bout duration (Fig 5). Thermogenetic activation of AstA^1^ and AstA^34^ cells significantly increased total sleep and sleep bout duration also under constant darkness (Fig 4D and 4E), and constant light conditions known to disrupt the clock (S9 Fig). Next we silenced AstA cells by constitutive expression of *UAS-Kir2*.*1* [30], yet without effect on activity or sleep (S10 Fig). However, when we conditionally silenced AstA cells using the TARGET system [53] and UAS-Kir2.1, sleep was significantly affected especially during the midday siesta time (Fig 6). This is in line with a significant increase in total activity (S11A Fig). A similar increase in locomotor activity and decrease in sleep upon UAS-Kir2.1 silencing was also observable in constant darkness, while rhythmicity and period of the locomotor rhythm was not affected (S12 Fig). An alternative neuronal silencer, UAS-ΔORK [60], did also not reduce sleep when constitutively expressed (S13 Fig). Under conditional expression, however, UAS-ΔORK lead to a significant increase in sleep only during the evening activity, and unexpectedly to decreased sleep during the early siesta time (S13 Fig).

**Fig 7:**
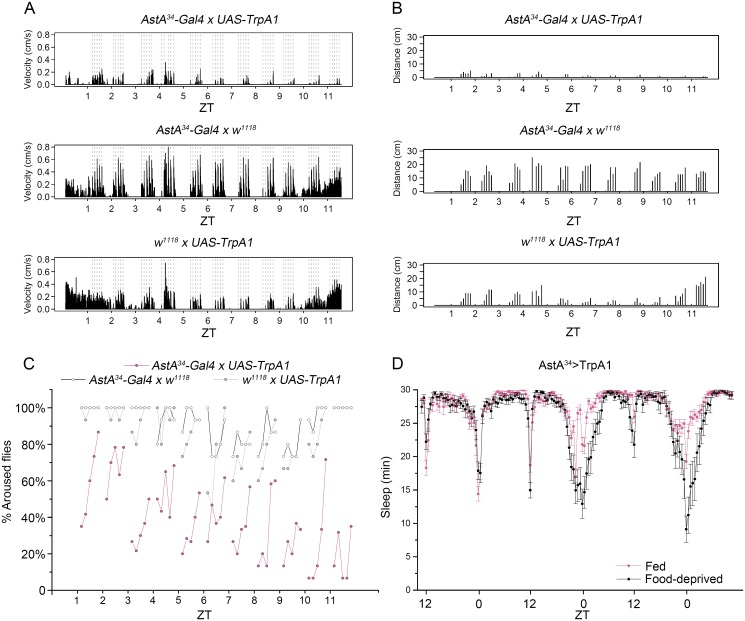
Mechanically- and starvation-induced activity. **(A-B**) An increasing level of mechanical stimuli by a loudspeaker (shown as dashed lines) were used to arouse flies in a glass tube (for details see material and methods). Thermogenetic activation of AstA cells in *AstA*^*34*^>*TrpA1* flies resulted in decreased average velocity **(A)** and a shorter distance walked **(B)** in a 2 min window after each stimulus compared to controls (n = 5). **(C)** An increasing level of mechanical stimuli were used to arouse flies in a Petri dish on a shaker (for details see material and methods). In general, the percentage of aroused flies increased with increasing shaking speed. While the arousal threshold for control flies seems to increase during the siesta phase during the middle of the day to decrease again towards the evening activity peak, there is a steady decline of the percentage of aroused flies during the day (n = 15). **(D)** Starvation-induced locomotor hyperactivity in flies with thermogenetically activated AstA cells reduces sleep during the morning and evening activity. Flies were kept at 20°C in LD12:12 on normal food, and then transferred to DAM glass tubes and switched to 29°C and feeding/starvation-conditions at ZT8 at the start of locomotor activity monitoring (n = 32).

To test for sleep intensity, we determined the arousal threshold during the day in two different assays (Fig 7). For the first assay, *AstA*^*34*^*>TrpA1* flies were put into glass tubes as used in the DAM monitor, and kept for three days at 29°C to thermogenetically activate AstA cells. On day four, the tubes were placed onto a loudspeaker at 29°C. Five separated 5Hz sine wave stimuli were given with increasing intensity every hour during the light phase from Zeitgeber Time 1 (ZT1) to ZT12, and velocity and distance walked for 2 min after each stimulus was measured. As expected [61], the arousal-related parameters were dynamic during the day and varied somewhat between genotypes in the controls (Fig 7A and 7B). Notwithstanding, *AstA*^*34*^>*TrpA1* flies walked on average significantly slower and covered less distance for all stimulus intensities and at all times during the light phase than controls (Fig 7A and 7B). Again, this phenotype is unlikely to be caused by impaired locomotor ability since the maximum speed reached by individual flies was similar between *AstA*^*34*^>*TrpA1* flies and controls (S14 Fig).

For the second assay, flies were put in small groups into Petri dishes and kept again for three days at 29°C. On day four, we monitored their activity in the Petri dishes placed on a shaker at 29°C during the light phase to better mimic the situation during the CAFE assay. The Petri dish was hourly agitated in a series of five 2s shakes with increasing speed separated by a 5 min break during which fly behaviour was manually analysed for the fraction of aroused flies after each stimulus. Again, control flies showed a dynamic arousal threshold that was higher during the afternoon "siesta" as expected (Fig 7C, S3 Movie), and a distinctly smaller percentage of aroused flies was observed for *AstA*^*34*^>*TrpA1* flies at all time points and intensities. Strikingly, the percentage of aroused flies was steadily decreasing during the course of the day and was lowest at the time of the evening peak activity (Fig 7C).

### Starvation decreases sleep in flies with activated AstA cells

Sleep and feeding are interconnected behaviours, and it is interesting to ask whether flies with activated AstA cells are prevented from eating more because their locomotor activity is reduced, leading to insufficient foraging activity although flies are "hungry". Alternatively, flies with activated AstA cells may eat less because they need less energy intake since they move less, and thus are "satiated". To find out which scenario applies, we monitored food intake in A*stA*^*1*^>*TrpA1* and *AstA*^*34*^>*TrpA1*flies that prior to the CAFE assay at 22°C had been kept under assay conditions for one day at 22°C and then for two days at 29°C to activate AstA signalling. Under these conditions, both *AstA*^*1*^>*TrpA1* and *AstA*^*34*^>*TrpA1* flies showed no feeding rebound after release from thermogenetical activation of AstA signalling (S15 Fig). This suggests that flies with activated AstA signalling are not in a hunger state, and further indicates that the observed feeding phenotype is not due to impaired locomotor ability. To test this further, we monitored the locomotor activity of fed (agarose with sugar) and starved (agarose without sugar) flies with thermogenetically activated AstA neurons. Wildtype flies respond to prolonged starvation with a phase of hyperactivity, interpreted as a hunger-driven food search [62,63]. Likewise, *AstA*^*34*^>*TrpA1* flies on starvation medium increased locomotor activity/reduced sleep compared to flies on food (Fig 7D). This provides further evidence that flies with activated AstA cells kept on food do not feel hungry. Off food, these flies become hungry as judged by their observed hyperactivity which argues against a general locomotor impairment in flies with activated AstA cells. The same phenotype was also seen with *AstA*^*1*^>*TrpA1* flies (S16 Fig). Obviously, the sleep-promoting effect of AstA neurons can at least partially be overcome by starvation, arguing against a direct dependence between the sleep-promoting and anorexic effect of AstA cells.

### Genetic distinction between AstA-expressing PLP neurons and EECs

So far, we could show that thermogenetic activation of AstA-signalling from PLP neurons and/or EECs inhibits feeding and promotes sleep. To distinguish between these AstA cell subsets, we next aimed to further restrict the thermogenetic activation to AstA EECs only, using panneuronal *elav-Gal80* [64]. To our surprise, *elav-Gal80* not only efficiently suppressed GFP expression in AstA neurons, but also in EECs (see S16C and S16D Fig). Since *elav* was reported to be specifically expressed in neurons and glia [65,66], we tested for *elav* expression in the midgut by immunostaining with an anti-ELAV monoclonal antibody which strongly and specifically stained EECs in the midgut (S17A and S17B Fig). A similar pattern was found when expressing GFP with an *elav-Gal4* driver line (S17E Fig). This indicates that the widely used panneuronal *elav-Gal4* drivers cannot be regarded as neuron/nervous system-specific, and suggests a role for *elav* in EEC differentiation. A second *Gal80* line used to restrict *Gal4* expression to the nervous system is *nsyb-Gal80* [67]. We found no *nsyb>GFP* expression in EECs, and tried to restrict *AstA*^*34*^*>GFP* expression to the EECs by co-expression of *nsyb-Gal80*. Co-expression of *Gal80* inhibited the expression of GFP in AstA neurons, but to our surprise also in the midgut EECs. In line with that, *nsyb-Gal80* completely suppressed the behavioural effects observed upon thermoactivation of the AstA^34^ cells (S18 Fig). These results caution against the assumption that *Gal80* patterns always fully replicate the respective *Gal4* pattern. *Prospero* is a EEC-specific marker for the gut [68,69], but expresses also broadly in the adult CNS [70] which prevented the use of *prospero-Gal4* for thermogenetic activation. Thus, we were unable to further genetically differentiate between PLP neurons and AstA EECs.

### The AstA-expressing PLP neurons are a direct target of the clock output factor PDF

**Fig 8:**
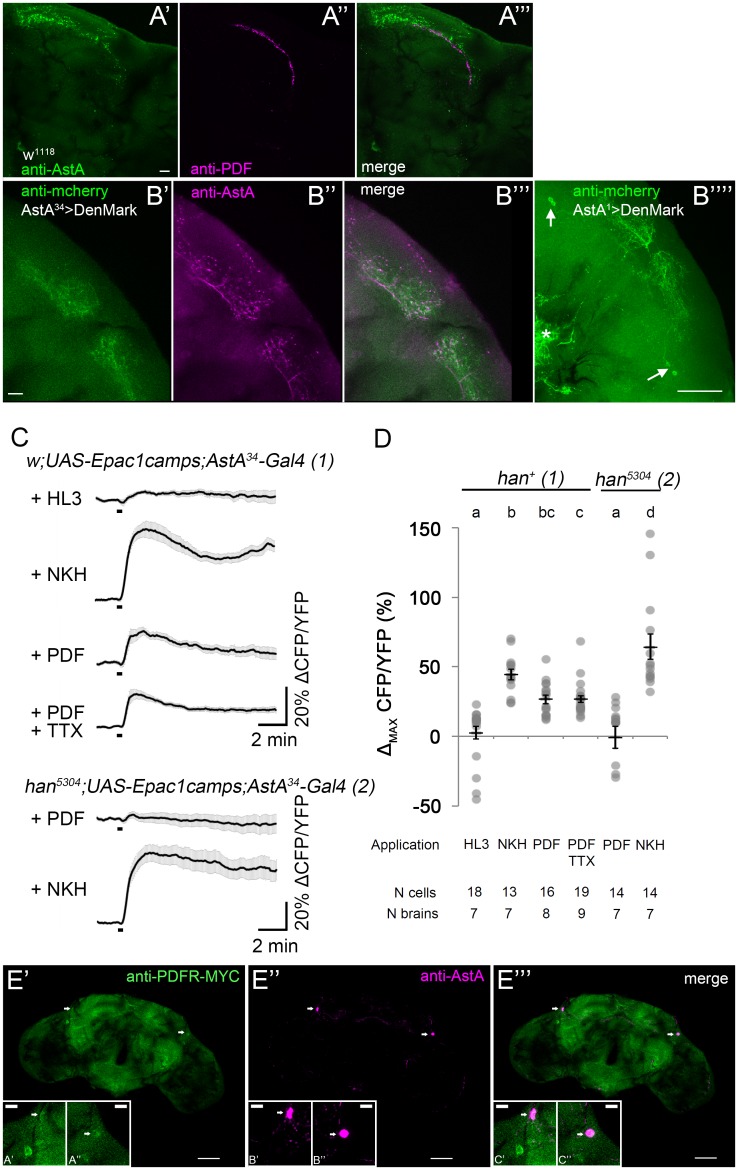
The AstA-expressing PLP neurons are a downstream target of the clock output factor PDF. **(A')** A 2 μm confocal section through the superior protocerebrum containing the arborisations of the PLP neurons immunolabelled against AstA. **(A'')** The same section contains the terminals of the PDF-expressing sLNv clock neurons visualised by immunostaining against PDF. **(A''')** Both peptidergic arborisations are in close apposition to each other. **(B)** The PLP arborisations in the superior protocerebrum are more extensively labelled by the postsynaptic marker UAS-DenMark (B') than by AstA staining **(B'')**. Fine branches that are only DenMark-labelled but AstA-negative are evident in the merged confocal section **(B''')**, suggesting that these branches represent input sites of the PLP neurons. **(B'''')** The DenMark labeling of PLP arborisations in a AstA1>DenMark brain extends over a large area in the superior protocerebrum and is not restricted to the site of contact with the sLNv. The asterisk marks DenMark-labeled dendrites of the AstA cells in the ventral brain/gnathal ganglia. Maximum projection of the PLP neurons. Scale bars: 10 μm, B'''' 50 μm.**(C-D)** *Ex vivo* live-cAMP imaging of central brain Allatostatin-A neurons. **(C)** Average inverse FRET traces (CFP/YFP) of Allatostatin-A neurons reflecting intracellular changes in cAMP levels. Substances were bath applied drop-wise between recording seconds 100 and 110 (black bar). Application of 10μM of the adenylate cyclase activator NKH477 led to a robust increase in cAMP, indicating that the general procedure was working. 10μM PDF peptide also evoked an increase in cAMP, indicating a functional connection between PDF expressing cells and PLP neurons. To test whether this functional connection was direct, brains were incubated in 2μM TTX for 15min prior to imaging and 10μM PDF were then coapplied together with 2μM TTX. The neurons responded with similarly increasing levels in cAMP, indicating that the signalling from the PDF neurons to the PLP neurons is not mediated by interneurons. **(D)** Maximum inverse FRET changes were quantified for each individual neuron (gray dots) and averaged for each pharmacological treatment (mean ± SEM: black horizontal lines). Statistical comparison revealed significant increases in cAMP levels compared to the negative control (HL3) for NKH477, PDF as well as PDF+TTX. Error bars represent SEM and letters indicate statistical significances. Statistics in D): Kruskal-Wallis H(3) = 39.507; Bonferroni-corrected Wilcoxon pairwise-comparison with negative control (HL3): NKH p = 0.006, PDF p = 0.018, PDF+TTX p<0.001; Bonferroni-corrected Wilcoxon pairwise-comparison with positive control (NKH477): PDF p = 0.066, PDF+TTX p = 0.012; Bonferroni-corrected Wilcoxon pairwise-comparison with PDF: PDF+TTX p = 1.0. (**E)** The PDFR expression marker PDFR-myc **(E')** is weakly expressed in the AstA-immunopositive (**(E'')** PLP neurons **(E''')**. Single confocal section. The inserts show the PLP somata in larger magnification. Scale bars: 50 μm, inserts 10 μm.

During our morphological analysis (Fig 1) we noticed that the PLP neurites in the superior protocerebrum make branches in the same area as the PDF-expressing small ventral lateral neurons (sLNvs), a main component of the central circadian clock. The neuropeptide PDF is a major synchronisation and output factor of the circadian clock [71] which affects the timing of sleep and feeding [18,72]. We therefore wanted to know whether the PLP neurons represent downstream targets of circadian PDF-signalling. Confocal microscopy first showed that indeed the projections of sLNvs and PLP neurons are overlapping in the superior protocerebrum (Fig 8A and 8B). While the sLNv projections represent mainly output sites [73], the PLP neurites seem to be postsynaptic as indicated by the expression of the postsynaptic marker DenMark:mcherry (Fig 8A and 8B). Using live cAMP imaging, we next asked whether PLP neurons express functional PDF receptors. Synthetic PDF was bath-applied to acutely isolated brains that expressed the cAMP sensor Epac-camps in the AstA^34^ neurons. A similar approach had previously been very successful to demonstrate functional PDF receptors on clock neurons [74]. The PLP neurons reacted with a fast increase in intracellular cAMP upon 10 μM PDF (Fig 8C and 8D), while control applications of saline had no effect. This PDF-mediated cAMP increase appeared to be by direct activation of PDF receptors on the PLP neurons since a similar cAMP increase was also seen after blocking neuronal conduction by tetrodotoxin (TTX, Fig 8C and 8D). PDF application had no effect on the PLP neurons in a PDF receptor mutant background (han^5304^ [75], Fig 8C and 8D). We also found that the PDFR expression reporter pdfr-myc [76] is weakly but consistently expressed in the PLP neurons (Fig 8E). Only very few further neurons in that area of the superior protocerebrum were weakly myc-positive; strongly myc-positive neurons comparable in staining intensity to the sLNvs were absent in that part of the brain. These results suggest that the PLP neurons represent downstream targets of circadian PDF signalling.

### Activation of AstA cells by tethered PDF increases sleep

To investigate the functional significance of PDF-PLP neuron signalling, we first aimed to down-regulate the expression of the PDF receptor by RNAi in AstA neurons. In preliminary test, however, none of the tested VDRC or Janelia PDFR RNAi-lines had an effect on circadian locomotor activity when expressed in PDF and other clock neurons (Pamela Menegazzi, pers. commun.), indicating a general lack-of-effect in these lines. We therefore switched to constitutive activation of PDF signalling by expressing membrane-tethered PDF (t-PDF) in AstA^34^ cells. A similar approach has been successfully used to study the sleep effects of calcitonin gene-related peptide/DH31 [77]. t-PDF activated co-expressed PDFR in heterologous cell culture and rescued rhythmicity when specifically expressed in clock neurons in a *pdf*^*01*^ mutant background [78]

**Fig 9:**
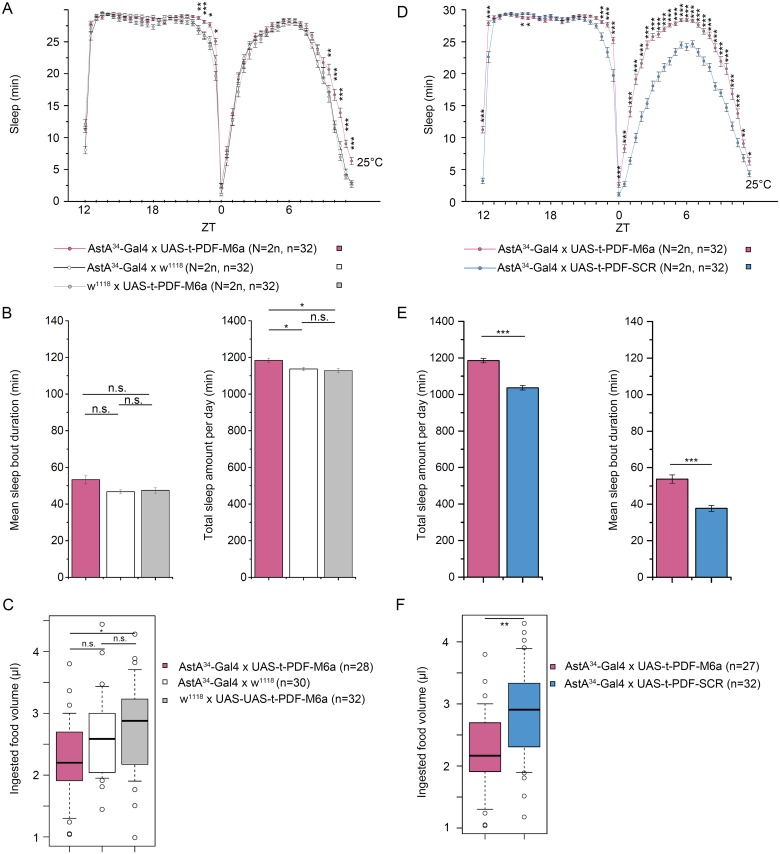
Effect of ectopic expression of tethered PDF (t-PDF) in AstA^34^ cells on sleep (A-B, D-E) and food intake (C,F). **(A-C)** Experiments using heterozygous controls. (**A**) t-PDF expression induced a small increase in sleep especially during the time of the evening activity. **(B)** Total sleep amount but not sleep bout duration was significantly increased by t-PDF expression. **(C)** t-PDF expression did not significantly reduce food intake over genetic controls. **(D-F)** Experiments using t-PDF-SCR as a control. (D) t-PDF expression induced increased sleep mostly during the light phase and lights-on anticipation compared to the AstA^34^>t-PDF-SCR control. (E) Quantification shows that mean sleep bout duration and the total amount of sleep was significantly increased by t-PDF expression. (**F**) t-PDF expression also significantly reduced food intake. * p ≤ 0.05. ** p ≤ 0.01, *** p ≤ 0.001.

When expressed in AstA^34^ cells, t-PDF induced a significant increase in total sleep compared to Gal4/UAS controls and flies expressing a scrambled non-functional version of t-PDF (Fig 9A, 9B, 9D and 9E). In accordance, total activity in *AstA*^*34*^*>t-PDF* flies was significantly reduced to about half of that of controls (S10B Fig). The effect of t-PDF expression, compared to Gal4/UAS controls, was most pronounced during the evening activity when no or little native PDF is released, and small during the peak time of native PDF release in the morning hours ([79,80], Fig 9A). Compared to flies expressing a scrambled version of PDFR, the effect of t-PDF expression was most pronounced during the light phase, and less pronounced during the dark phase when also the PDFR-SCR control flies slept most of the time (Fig 9D and 9E).

This suggests that t-PDF-induced PDFR signalling activates AstA^34^ cells, in line with the reported activating effect of t-PDF on sLNvs [81] and that this ectopic activation is most effective when native PDF release is absent. The timing of the activity peaks was unaltered. In addition, *AstA*^*34*^*>t-PDF* flies fed significantly less than the PDFR-SCR and UAS-TRPA1 control (Fig 9C), again in line with the notion that t-PDF increases the activation of AstA^34^ cells. No significant difference, however, was detectable for the AstA^34^-Gal4 control. We note that for the t-PDF-SCR expressing flies the total amount of sleep and the sleep bout duration was considerably lower than for other controls (Fig 9B), mostly due to a low amount of sleep during the day (Fig 9A).

**Fig 10:**
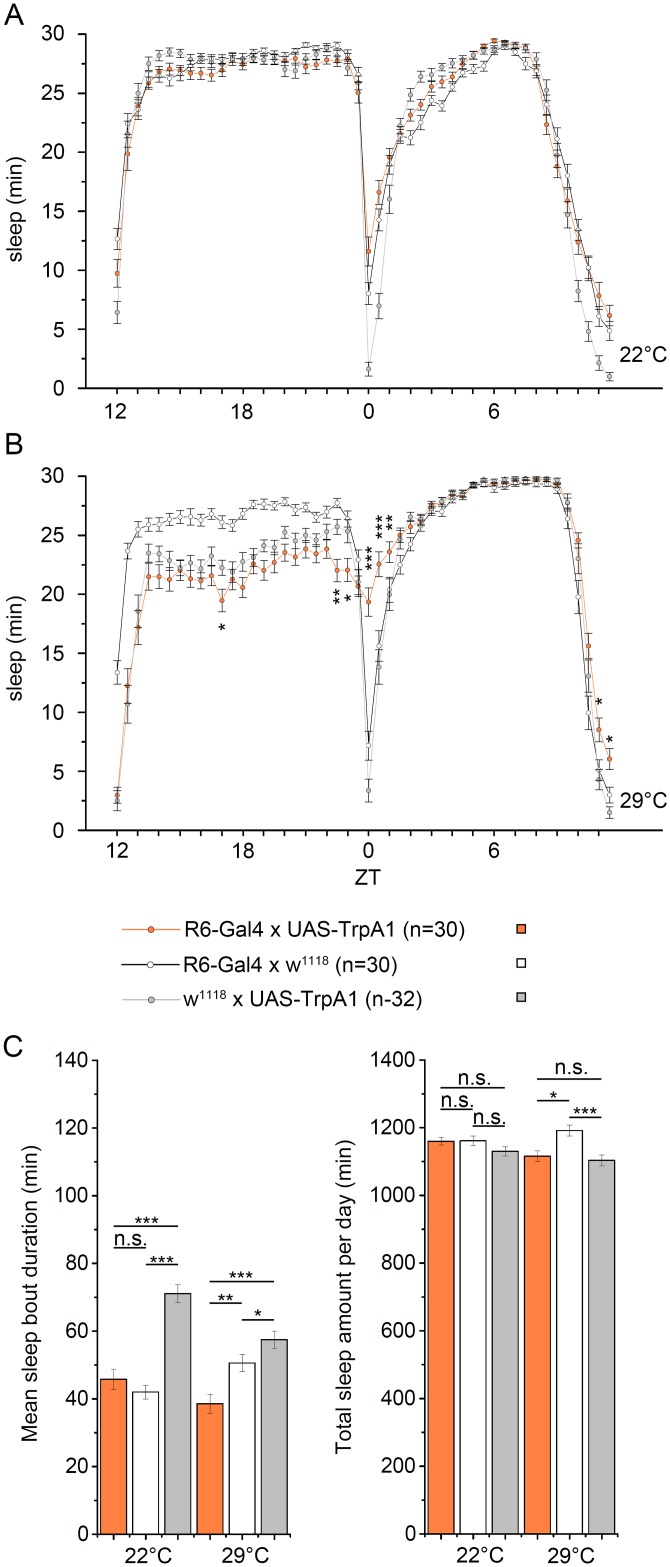
Activation of the PDF-expressing sLNvs promotes sleep specifically during the time of morning and evening peak activity. **(A)** At 22°C, *R6*>*TrpA1* flies showed the same sleep pattern than controls. Activation of the TrpA1 channel at 29°C resulted in increased sleep time specifically during the time of the morning and, to a lesser amount, the evening activity **(B)**. (**C)** Both mean sleep bout duration and the total amount of sleep per day was not affected by activation of the sLNvs.

The observed changes in sleep after expression of t-PDFR are considerably smaller but go in the same direction than the changes observed upon activation of AstA^34^ cells (Fig 5), suggesting that PDF positively modulates rather than strongly activates AstA^34^ cell activity. To test this assumption, we thermogenetically activated the PDF-expressing sLNvs using the R6-Gal4 driver line [82]. As expected if PDF activates AstA cells, activating sLNvs increased sleep and not activity. Yet, the effect was limited to the time of morning and evening peak activity and was -again- much smaller compared to thermogenetic activation of the AstA cells (Fig 10A). Total sleep and sleep bout duration over the day was not significantly altered (Fig 10A''').We cannot exclude that this mild effect is at least in part caused by co-activation of one or two large LNvs which weakly express R6-Gal4 [83] and have been shown to promote arousal [72].

Based on these results and the anatomical and imaging data, we conclude that PDF from the sLNvs positively modulates PLP neurons without affecting the phase and general timing of AstA-regulated behaviours.

## Discussion

Our study shows that AstA cells via AstA signalling subserve an anorexigenic and sleep-promoting function in *Drosophila*. In mammals, a variety of neuropeptides and peptide hormones affect both sleep and feeding [4,5], and our results provide evidence that also further such peptides exist in the fly besides sNPF and possibly NPF [21,24,25]. More specifically, our results with a new *AstA*^*34*^*-Gal4* driver line show that activation of AstA-expressing PLP brain neurons or numerous EECs in the midgut strongly reduces food intake and promotes sleep. These behavioural effects are congruent with the anatomy of these cells. PLP interneurons are well positioned to modulate sleep as they widely arborise in the posterior superior protocerebrum, a projection area of sleep-relevant dopaminergic neurons [84,85], superior (dorsal) fan-shaped body neurons [86–88] and neurons of the pars intercerebralis [89]. AstA EECs in *Drosophila* are “open type” EECs [31,32], possessing apical extensions that reach the gut lumen and likely express gustatory receptors [90]. AstA-expressing EECs are thus potentially able to humorally signal nutritional information from the gut to brain centres regulating feeding and possibly also sleep and locomotor activity. If AstA is involved in inhibiting feeding and promoting sleep, one could expect AstA mutants to display decreased sleep and increased feeding in the absence of any other manipulation of AstA cells. We observed, however, that a functional loss of the *AstA* gene did neither affect feeding nor locomotor activity under the experimental conditions with unrestricted access to a food source. This may suggest that AstA signalling is not part of a core feeding network, but represents an extrinsic modulator which becomes activated under specific yet so far uncharacterised conditions. Alternatively, as suggested by the observed difference in effect of constitutive vs. conditional electrical silencing of AstA cells, flies may be able to genetically or neuronally compensate for a constitutive loss of AstA signalling during development.

In larval *Drosophila*, AstA inhibits midgut peristalsis and affects K^+^ transport [35] in order to concentrate ingested food. Together with our finding of a sleep-promoting and feeding-inhibiting effect of AstA, we propose that pleiotropic AstA signalling serves to coordinate behaviour and gut physiology to allow for efficient digestion. After food intake, AstA from the PLP neurons or EECs cause inhibition of further feeding, and -as the need for food search behaviour is relieved and nutrients need to be taken up- promotes sleep and inhibits gut peristalsis. Based on the gut content, enteroendocrine AstA is released and hormonally activates DAR-2 on key metabolic centers to tune adipokinetic hormone and insulin signalling [91], and -at least in other insects- stimulates digestive enzyme activity in the midgut [92,93].

The AstA receptors are homologues of the vertebrate galanin receptors [40–43] that have pleiotropic functions [44]. When activated in specific brain areas, galanin signalling has a strong orexigenic effect [45] and has also been implicated in the control of arousal and sleep in mammals [45]. In zebrafish, transgenic heat-shock induced expression of galanin decreased swimming activity, the latency to rest at night and decreased the responsiveness to various stimuli [94]. Furthermore, the allatostatin/galanin-like receptor NPR-9 inhibits local search behaviour on food in the nematode *C*. *elegans* [95]. Similar to AstA in *Drosophila* [35], galanin modulates intestinal motility and ion transport [44]. Thus, in broad terms, the involvement of DARs/galanin receptors in modulating feeding, gut physiology and arousal/sleep appears to be evolutionarily conserved.

The neuronal clock network in *Drosophila* is intrinsically and extrinsically modulated by a variety of peptides (sNPF, NPF, calcitonin-gene related peptide/DH31, ion transport peptide, myoinhibiting peptides and PDF), which all affect sleep and locomotor activity and in part also act as clock output factors [24,77,96–100]. Our imaging results and constitutive activation of the PDF signalling pathway by t-PDF now suggest that the PLP neurons are modulated by PDF originating from the sLNv clock neurons. Unlike the peptides above, AstA from PLP neurons is outside and downstream of the central clock and seems not to modulate the clock network. Due to their anatomy and position, PLP neurons thus appear well-suited candidate cells by which clock neurons could modulate the complex cross-regulatory network regulating sleep, locomotor activity and perhaps also feeding. The rather mild effects on sleep and feeding of either t-PDF expression in AstA cells or thermogenetic activation of the sLNvs implies that this pathway is not the major output target of the central clock (if there is any) to modulate feeding and locomotor activity/sleep. We found no shift in the circadian period or phase of feeding and locomotory activity/sleep upon AstA cell activation, suggesting that the main function of PDF-to-AstA cell signalling is not to time the respective behaviours but to modulate their amplitude. Similar non-timing functions of PDF have been demonstrated for other behaviours, including geotaxis and rival-induced mating duration [101,102].

At first sight, our data suggesting that PDF activates PLP neurons to promote sleep seem to contradict earlier findings [72]. Since pdf^01^ mutants show increased sleep during the photophase, the arousal effect appears to be the dominant effect of PDF which is due to signalling between ventral lateral clock neurons (LNvs) [72], with a major contribution of the PDF-expressing large LNvs [103]. The PLP neurons are only contacted by the sLNvs, which upon activation induced a time-specific increase in sleep, but did not increase arousal. Thus, the sLNv-PLP pathway likely represents a sleep-promoting clock output branch. Besides PDF, the sLNvs but not the lLNvs also co-localise the sleep-promoting peptide sNPF [24]. A recent report shows that hormonal PDF released from abdominal PDF neurons serves to couple the central clock with a peripheral clock in the oenocytes [104]. Furthermore, the posterior midgut is innervated by the abdominal PDF neurons [32], and PDFR is expressed in the midgut [105]. It is thus possible that the AstA-expressing EECs represent additional PDF targets and may contribute to the PDF-related effects of AstA cells.

In conclusion, the lack of effect on feeding upon AstA cell silencing under non-restricted food availability and an unaltered circadian locomotor rhythmicity after AstA cell silencing suggests that AstA signalling is neither a primary signal in feeding regulation nor in the clock output pathway timing rhythmic behaviour. Rather—like mammalian galanin signalling [45]- it seems to be one out of several modulatory pathways that allow to adapt the intensity of feeding and locomotor activity/sleep to specific physiological or environmental conditions. For example, decreased locomotor activity to save energy and increased digestion efficiency to maximise energy uptake may be most important during restricted food conditions, at which AstA cell silencing leads to increased feeding [28]. While our results allow now to raise such speculations, it is clear that more research is needed to reveal the conditions at which AstA signalling is functional and the modulatory PDF input is strongest.

## Materials and Methods

### Flies

Following strains were used: *w;AstA*^*1*^*-Gal4 [28]*, kindly provided by D. Anderson, Caltech, CA, USA), *w;tsh-Gal80/CyO* (kindly provided by J. Simpson), *elav-Gal4* (Bloomington Stock Center), *elav-Gal80* [64], kindly provided by LY and YN Jan), *w*;*nsyb-Gal80* [67], kindly provided by Stephen F. Goodwin—originally from J. Simpson), *prospero-Gal4* (kind gift of J. F. Ferveur), *nsyb-Gal4* (kindly provided by T. Langenhan), *386y-Gal4* and *w;;Pdfr-myc* ([76,106], kindly provided by Paul Taghert), *UAS-Dcr-2* (VDRC Stock #60007) *UAS-AstA-RNAi* (VDRC Stock #103215 KK), *w;UAS-DenMark* [107], kind gift of Bassem Hassan)*, UAS-Epac1camps* [74], *10xUAS-IVS-myr*::*GFP* ([108], Bloomington Stock Center), UAS-tethered-PDF (UAS-t-PDF-M6a containing one transgene copy, and control UAS-t-PDF-SCR A2 [78], kindly provided by Joel Levine), *w;UAS-TrpA1* (Bloomington Stock Center), *w;UAS-Kir2*.*1* ([30], *w;;UAS-ΔOrk-ΔC1* and *w;;UAS-ΔOrk-ΔNC1* ([60], *Bloomington Stock center)*,*UAS-tubGal80*^*ts*^ [53] *han*^*5304*^ ([75]and *Canton-S* wildtype and *w*^*1118*^ for control crossings (all from Bloomington Stock Center). Flies were kept on standard *Drosophila* medium (Supplementary file 1) at a 12:12 h light-dark cycle (LD, in which lights-on is defined as ZT0 and lights-off as ZT12) and 25°C, except for the crossings used in TrpA1 experiments, which were kept at 20 or 22°C.

### Creation of AstA promoter-Gal4 transgenic flies

The putative *D*. *melanogaster* allatostatin A promoter region was amplified from genomic DNA by PCR using three different primer sets that amplified 1.03 kb, 2.05 kb and 2.74 kb upstream of the transcription initiation site (see S1 Text). The resulting PCR products were cloned into pCR-TOPO. The inserts were digested with *Mun*I and *Bam*HI, gel purified and exchanged with the *Akh* promotoer in the *pAkh-Gal4* vector [109]. The resulting *P{pAstA-Gal4}* plasmids were injected into *Drosophila* embryos by BestGene Inc. (Chino Hills, CA, USA) and at least 5 independent P-element transformant lines per construct were obtained. While the short 1.03 kb promoter fragment failed to direct GAL4 expression to AstA-immunoreactive (IR) cells, longer 2.05 and 2.74 kb promoter fragments lead to GAL4 expression in varying subsets of AstA-IR cells in the larval CNS and midgut (S1 Fig). We chose the 2.74 kb promoter line *AstA*^*34*^-*Gal4* for our experiments, since it showed the most restricted and AstA-specific cellular distribution among the different 2.74 kb promoter lines generated by P element transposition (S1 Fig).

### Creation of AstA mutant flies

The generation of AstA mutants by germline-specific expression of Cas9 and guide RNA (gRNA) transgenes [57] was already described [91]. Mutant stocks were established from two alleles, AstA^SK1^(used by [91]) and AstA^SK4^ (w^1118^;;AstA^SK4^ used throughout this study), in which the start codon of the AstA gene is removed.

### Immunostaining

Tissue of feeding 3^rd^ instar larvae or adult flies (approx. 1 week after eclosion at 25°C) was dissected in HL3.1 solution [110] and fixed in 4% PFA/PBS (pH 7.2) at room temperature for 45 min (guts and larval CNS) or 90 min (adult CNS). After several washes with PBT (= PBS with 0.3% Triton X) followed by an overnight blocking step with PBT containing 10% normal goat serum at room temperature, the tissue was incubated in primary antibody solution on a shaker for 1d at 4°C, then several hours at room temperature. Primary antibodies were diluted in PBT containing 3% normal goat serum. 1d of washing steps with PBT followed, after which the samples were incubated with secondary antibodies diluted 1:300 in PBT containing 3% normal goat serum. Samples were again washed several times with PBT, then twice with PBS and finally mounted onto microscope slides using 80% glycerol/20% PBS. Images were acquired with a Leica TCS SPE or SP8 confocal microscope (Leica, Wetzlar, Germany). Fiji [111] was applied for maximum intensity projection and contrast enhancement. Figures were generated with Adobe Photoshop CS2.

Primary antibodies used were a mouse anti-GFP IgG mAb (1:100, A11120, Invitrogen GmbH, Karslruhe, Germany), a rat anti-ELAV mAb (1:100, 7E8A10, developed by GM Rubin, obtained from the Developmental Studies Hybridoma Bank, University of Iowa, IA, USA), monoclonal rat anti-mCherry (1:1000, Molecular Probes, Frederick MD, USA), mouse anti-Myc-tag mAb (1:1000, 9B11 mouse, mAb New England Biolabs, Frankfurt, Germany) and a polyclonal rabbit antiserum directed against Dippu-AstA-7 (1:2000, [112], Jena Bioscience GmbH, Germany) which recognizes the C-terminal YXFGL-amide of AstA peptides including that of Drome-AstA-1–3 [113]. Alexa Fluor 532 and 647- or DyLight 488-conjugated IgG (H+L) secondary antibodies were purchased from Dianova GmbH, Hamburg, Germany.

### Capillary Feeder (CAFE) assay

The CAFE protocol followed [47]. 4–5 old male or female flies were anesthetized on ice and transferred into 24-well plates (1 fly per well) containing several small holes in each well to allow for air exchange. A piece of moist filter paper was added to each well to provide the flies with water separately from the food. Capillaries (5 μl glass capillary pipettes, Megro GmbH & Co. KG, Wesel, Germany) were filled with liquid food and one capillary per well was inserted through a hole in the lid of the well plate so that the bottom was easily accessible to the fly. Food capillaries in wells without flies were used to control for evaporation. The amount of evaporated liquid in these control capillaries was substracted from the other capillaries in the capillary assays. The plates were put into an airtight, humid container and placed into an incubator with a 12:12 h LD at 22°C or 29°C. Liquid food was prepared fresh every day and contained: 5.4% sucrose, 3.6% yeast extract (BioChemica, AppliChem, Darmstadt, Germany) and 0.03% BPB (Bromophenol blue sodium salt, electrophoresis grade, AppliChem, Darmstadt, Germany) (all m/v) in ultrapure water. Capillaries were exchanged each day at the same time. Food consumption was not measured for the first day to give the flies some time to acclimatize to the change of environment and food. Values measured for day 2 and 3 (descent of the meniscus) were summed up for each fly.

### Startle-induced negative geotaxis assay

Four to five days old flies of each genotype were kept for 24h at 22°C on normal food, 29°C with normal food or at 29°C with water only. For each trial, 10 male flies were transferred to a 50ml falcon tube. These tubes were tapped gently on the table, and the number of flies which climbed over an 8 cm marker within 10 seconds was calculated. For each experiment, this was repeated 10 times.

### Locomotor activity and sleep measurement

Drosophila Activity Monitors (DAM, TriKinetics Inc., Waltham, MA, USA) were used to measure locomotor activity. 4–5 days old adult males or females were transferred to separate glass tubes containing an agar-sucrose food medium (prepared from 2% agar and 4% sucrose in ultrapure water by brief boiling), after which the tubes were closed with foam plugs. The tubes were inserted into holes in the monitor and centered. As a fly walked back and forth within its tube, it interrupted an infrared beam that crossed the tube at its midpoint. Light beam interruptions were counted for individual flies at 1-min intervals as a measure of fly activity. Flies were monitored under a 12:12 h LD with 365 lux light intensity at 22°C and subsequently at 29°C; average minute-by-minute activities during the day were calculated for both conditions. Activity and sleep data was analysed using ActogramJ [114] and a custom-made Excel macro by Taishi Yoshii [115].

### Arousal assay

Flies were kept in a 29°C incubator, LD12:12, for 3d prior to the experiments. Two different arousal setups were used, with flies kept in tubes or Petri dishes, respectively.

Tube assay: During the assay, each fly was individually housed in a 65mm glass tube (Trikinetics). For each experiment, we used 5 tubes for each genotype, which were laid on a loudspeaker (VISATON WS 25E, 8Ω). On the 4th day from ZT1 to ZT12, stimuli of increasing intensity between 0.4 and 2.0 volt (steps of 0.4 Volt) were consecutively delivered and behaviour was recorded by a camera (PENTAX TV LENS 25mm 1:1.4) at 1 Hz using IC Capture 2.2 software. Stimuli were generated with a PHILIPS PM 5139 function generator coupled to a TAURUS A2100 stereo amplifier and a loudspeaker to generate a 5Hz sine wave to wake up the flies. The interval between the individual stimuli was between 5–8 min. Average walking velocity (cm/s) and stimulus-induced walking distance for a 2 min window after each stimulus was analysed with MetaMorph version 7.8.0 (Molecular Devices, Sunnyvale, CA, USA) from ZT1 to ZT 12.

Petri dish assay: 5 flies were housed in a Petri dish filled with 2% agarose containing 4% sucrose on a shaker (Edmund Bühler KL-2, Tübingen, Germany). On the 4th day from ZT1 to ZT 12, five mechanical shakes with increasing speed (50, 100, 200, 300 and 400 rpm) were delivered for 2 seconds. The interval between the individual stimuli was between 5–8 min. Fly behaviour was recorded at 1 frame/s for 12h and locomotor activity was measured by eye, then arousal thresholds (percentage of flies moving) were calculated for each stimulus.

### cAMP live imaging

The ratiometric cAMP sensor UAS-Epac1camps [116] was expressed under the control of the *AstA*^*34*^*-Gal4* driver line. Homozygous 5–7 days old male *w;UAS-Epac1camps*;*AstA*^*34*^*-Gal4* flies were freshly dissected in cold Hemolymph-like saline (HL3 [117]) and isolated brains were mounted with the posterior surface up on the bottom of a Petri-dish containing HL3. Brains were allowed to recover from dissection for 15min prior to imaging. Live-imaging was conducted using an epifluorescent imaging setup (Zeiss AxioExaminer D1, Specta-X hybrid solid state LED source, or a VisiChrome High Speed Polychromator System with a ZEISS Axioskop2 FS plus, Visitron Systems GmbH, Puchheim, Germany) equipped with a 40x dipping objective (Zeiss 20x/1,0 DIC M27). Central brain AstA neurons were brought into focus and regions of interest (ROIs) were defined on single cell bodies using the Visiview Software (version 2.1.1, Visitron Systems, Puchheim, Germany). Time lapse frames were imaged with 0.2Hz by exciting CFP. CFP and YFP emissions were separately recorded with sCMOS cameras (pco.edge 4.2., PCO AG, Kelheim, Germany, connected via a Cairn TwinCam) or with a CCD-camera (Photometrics, CoolSNAP HQ, Visitron Systems GmbH using a beam splitter). After measuring baseline FRETs for 100s, substances were bath applied drop-wise between recording seconds 100 and 110. PDF peptide was synthesized by Iris Biotech GmbH (Marktredwitz, Germany) and was applied in a concentration of 10μM in 0.1% DMSO in HL3. The water-soluble forskolin derivate NKH477 served as positive control in a concentration of 10μM, while HL3 alone was applied as negative control. Both negative and positive controls also contained 0.1% DMSO. For tetrodotoxin (TTX) treatments, brains were incubated for 15min in 2μM TTX in HL3 prior to imaging and substances were coapplied together with 2μM TTX (as described in [118]). Intensity data for CFP and YFP emissions of each ROI were exported into Excel and inverse FRET (iFRET) was calculated over time according to the following equation: iFRET = CFP/(YFP-CFP*0.357) [74]. Thereby, raw CFP and YFP emission data were first background corrected and YFP data were further corrected by subtracting the CFP spillover into the YFP signal, which was determined as 35.7% of the CFP signal. Individual neuronal traces were finally normalized to baseline and were averaged for each treatment. Maximum iFRET changes were quantified for each individual neuron, then averaged for each pharmacological treatment and statistically compared.

### Statistics

Plotting and statistical analysis were performed using OriginPro 9.1G and the R environment (http://www.r-project.org/). One-way ANOVA with post-hoc Tukey's HSD tests were applied if criteria for normal distribution (Shapiro-Wilk normality test, p > 0.05) and homogeneity of variances (Levene's test, p > 0.05) were met, otherwise Kruskal-Wallis and post-hoc Mann-Whitney U tests (with Holm correction) were applied. Exceptions are stated in the figure legends.

## Supporting Information

S1 Text Recipe for standard Drosophila medium and primers. (DOC)

S1 Table Expression patterns of AstA^34^-Gal4 and tsh-Gal80; AstA^34^-Gal4. (DOC)

S1 Fig UAS-LacZ expression pattern in the larval CNS in relation to AstA immunostaining for the different generated *AstA-Gal4* lines. AstA-LacZ overlap: *Gal4-UAS-LacZ* expressing cells are AstA-immunpositive, AstA-LacZ false positives: *Gal4-UAS-LacZ* expressing cells are not AstA-immunopositive, AstA-LacZ false negatives: AstA-immunopositive cells not contained in the *Gal4-UAS-LacZ* expression pattern. The following primer sets were used to amplify the respective promoter regions:
pAstA1:
5’-GCGCAATTGATGGCTATTTCCCAGCTCCT-3’
5’-GCCGGATCCAGAGGTTCCGCGGACTAAAT-3’
pAstA2:
5’-GCGCAATTGAGTAGAAGCTGCGCCAGAAG-3’
5’-GCCGGATCCAGAGGTTCCGCGGACTAAAT-3’
pAstA3:
5’-GCGCAATTGGGGAAAAATCTCCGAAAACC-3’
5’-GCCGGATCCAGAGGTTCCGCGGACTAAAT-3’
(TIF)

S2 Fig Schematic summary of the expression pattern of the two *AstA-Gal4* drivers used, in conjunction with *tsh-Gal80*. AstA neurons in the posterior lateral protocerebrum (PLP cells) are in red, the dorsolateral abdominal AstA neurons (DLAa) in the thoracico-abdominal ganglion and the peripheral neurons (PN) are in blue, other AstA neurons in the ventral brain (VBN) and lateral optic lobes (LOL) are in green. AstA-expressing enteroendocrine cells (EECs) in the posterior midgut are represented by red triangles.
(TIF)

S3 Fig Thermogenetic activation of the AstA cells of female adults resulted in reduced food intake. **(A)** *AstA*^*1*^*>TrpA1*. **(B)** *AstA*^*34*^*>TrpA1* and respective controls. * p ≤ 0.05, ** p ≤ 0.01, *** p ≤ 0.001.
(TIF)

S4 Fig Conditional silencing of AstA^34^ cells by temperature-dependent expression of Kir2.1 did not alter food consumption: *tubGal80*^*ts*^;*AstA*^*34*^*>Kir2*.*1* flies with silenced AstA cells at 29°C and with normally active AstA cells at 18°C consumed the same amount of food then the controls. (TIF)

S5 Fig AstA RNA-interference rescued reduced food intake but not locomotor activity in flies with thermogenetically activated AstA cells. At 22°C, food intake (**A**) and locomotor activity (**B**) of *AstA*^*34*^*>TrpA1* flies did not show significant differences to controls. At 29°C, thermogenetic activation of AstA^34^ cells resulted in lower food intake (**A**) and locomotor activity (**B**). *AstA*^*34*^*>TrpA1/UAS-dcr-2*; *AstA-RNAi* flies were not significantly different in food consumption to *AstA*^*34*^*>TrpA1*, but showed a significantly reduced locomotor activity. * p ≤ 0.05. ** p ≤ 0.01, *** p ≤ 0.001.
(TIF)

S6 Fig Thermogenetic activation of AstA cells resulted in a strongly inhibited locomotion. Examples of double-plotted single fly actogramms underlying the results shown in Fig 4. Flies were initially kept at 22°C, then temperature was raised to 29°C at the time point indicated by a red arrow.
(TIF)

S7 Fig Thermogenetic activation of AstA^1^ cells reduced food intake and locomotion in the CAFE assay. Individual flies were filmed for 4 hours in a modified CAFE assay (three flies per genotype). Behaviour was categorized as "not moving", "moving" and "feeding". *AstA*^*1*^*>TrpA1* flies with activated AstA^1^ cells moved and consumed less than controls.
(TIF)

S8 Fig Thermogenetic activation of AstA cells strongly promoted sleep also in female flies. At 20°C, *AstA*^*34*^>*TrpA1* (top left) and *AstA*^*1*^>*TrpA1* females (bottom left) did not sleep more than controls. Activation of the TrpA1 channel by 29°C resulted in increased sleep time of *AstA*^*34*^>*TrpA1* (top right) and *AstA*^*1*^>*TrpA1 (*bottom right) females during the light phase from ZT0 to ZT12.
(TIF)

S9 Fig Thermogenetic activation of AstA^1^ cells (A-B) or AstA^34^ cells (C-D) increased sleep under LL conditions known to impair the clock and to induce arrhythmicity. (TIF)

S10 Fig Constitutive silencing of AstA cells by ectopic expression of Kir2.1 did not alter sleep behaviour in *AstA*^*34*^*>Kir2*.*1* **(A)** and *AstA*^*1*^*>Kir2*.*1* **(B)** flies. The mean sleep bout duration **(C)** and the total amount of sleep per day **(D)** of *AstA*^*34*^*>Kir2*.*1* and *AstA*^*1*^*>Kir2*.*1* is not significantly different to all controls.
(TIF)

S11 Fig **A)** Conditional silencing of *AstA*^*34*^ cells increases the mean locomotor activity. **B)** Ectopic expression of t-PDF in AstA^34^ cells decreases the mean locomotor activity.
(TIF)

S12 Fig Conditional silencing of AstA^34^ cells by UAS-Kir2.1 decreased sleep in constant darkness (DD). **(A)** Actogramms of single flies show that locomotor activity increases during the subjective day and night upon silencing of AstA^34^ cells. Rhythmicity and period is not affected. Both the duration of sleep bouts **(B)** and total sleep **(C)** is reduced.
(TIF)

S13 Fig The effect on sleep of constitutive **(A-B)** and conditional **(C-E)** silencing of AstA^34^ cells by UAS-ΔORK under LD12:12 **(A-B)** Constitutive silencing let to a slight increase in the total amount of sleep **(B)**, mostly due to increased sleep during the day **(A)**. This effect is opposite of the expected decrease upon AstA cell silencing. **(C-E)** Conditional silencing did not affect total sleep or sleep bout duration **(E)**, yet sleep is increased during the end of the evening activity and decreased during the first half of the photophase.
(TIF)

S14 Fig Velocity of flies after mechanical arousal by a loudspeaker at 29°C (compare to Fig 7 which shows the average velocity of several flies). While *AstA*^*34*^>*TrpA1* flies walked less (leading to a reduced average velocity), the maximum speeds when moving where not different to control flies, suggesting that the reduced locomotor activity is not due to motor impairment.
(TIF)

S15 Fig Lack of a feeding rebound after releasing the activation of AstA cells. *AstA*^*34*^>*TrpA1* and *AstA*^*1*^>*TrpA1* flies were kept for 1 day at 22°C, then 2 days at 29°C in the CAFE assay. Afterwards, flies were put back again to 22°C, and food consumption was summed up for the first 3, 6 and 24h.
(TIF)

S16 Fig Starvation-induced locomotor hyperactivity in flies with thermogenetically activated AstA^1^ cells reduces sleep especially during morning activity. Flies were kept at 20°C in LD12:12 on normal food, and then transferred to DAM glass tubes and switched to 29°C and feeding/starvation-conditions at ZT8 at the start of locomotor activity monitoring (n = 32).
(TIF)

S17 Fig Expression pattern of ELAV, *elav-Gal80* and *elav-Gal4* in L3 midguts. **A** and **B**): GFP expression (anti-GFP staining, green in **A'/B'**) driven by the peptidergic cell marker *386y-Gal4* (Taghert et al. 2001, Reiher et al. 2011) colocalises with a-ELAV immunoreactivity (magenta, **A''/B''**) as seen in the merged pictures A'''/B'''. Scale bar = 50 μm. **C** and **D**): *386y-Gal4* driven GFP expression **(C)** is suppressed by co-expression of *elav-GAL80* **(D)**. Widefield pictures taken with a CCD camera with an exposure time of 3.75 s (C'), 2.5 s (C''), 5 s (D') and 7.5 s (D'). All other camera settings were kept constant. **E**): *elav-Gal4*-driven native GFP expression in EECs. Arrows point to neurons in the proventricular ganglion.
(TIF)

S18 Fig *nsyb-Gal80* rescued reduced food intake and locomotor activity. Thermogenetic activation of AstA^34^ cells resulted in significant lower food consumption **(A)** and locomotor activity **(B)** compared to controls. Food intake (**A**) and locomotor activity (**B**) of *nsyb-Gal80; AstA*^*34*^*>TrpA1* were not significantly different to controls, but significantly higher than *AstA*^*34*^*>TrpA1* at 29°C. * p ≤ 0.05. ** p ≤ 0.01, *** p ≤ 0.001.
(TIF)

S1 Movie 3D rotation of the PLP neuron arborisations in the dorsal protocerebrum, anti-GFP staining in an adult *AstA*^*34*^*>GFP* fly. (AVI)

S2 Movie 3D rotation of AstA neuron immunoreactivity in the dorsal protocerebrum, same brain as in Suppl. video 1. (AVI)

S3 Movie The behaviour of *AstA*^*34*^*>TrpA1* (top), *AstA*^*34*^ *x w*^*1118*^ (right) and *w*^*1118*^ *x UAS-TrpA1* (left) flies upon increasing mechanical stimuli in the shaker assay at ZT1 to ZT12. (MP4)
