# Correction: Allatostatin A Signalling in *Drosophila* Regulates Feeding and Sleep and Is Modulated by PDF

**DOI:** 10.1371/journal.pgen.1006492

**Published:** 2016-12-06

**Authors:** Jiangtian Chen, Wencke Reiher, Christiane Hermann-Luibl, Azza Sellami, Paola Cognigni, Shu Kondo, Charlotte Helfrich-Förster, Jan A. Veenstra, Christian Wegener

There are errors in the fourth paragraph of the subsection ‘Activation of AstA cells by tethered PDF increases sleep’ in the Results. The following sentences are incorrect: “Yet, the effect was limited to the time of morning and evening peak activity and was -again- much smaller compared to thermogenetic activation of the AstA cells (Fig 10A). Total sleep and sleep bout duration over the day was not significantly altered (Fig 10A'').” These sentences should read: “Yet, the effect was limited to the time of morning and evening peak activity and was -again- much smaller compared to thermogenetic activation of the AstA cells (Fig 10A and 10B). Total sleep over the day was not significantly altered (Fig 10C).”

There are errors in the caption for Fig 10, “Activation of the PDF-expressing sLNvs promotes sleep specifically during the time of morning and evening peak activity,” panel C. Please see the complete, correct Fig 10 caption here.

**Fig 10:**
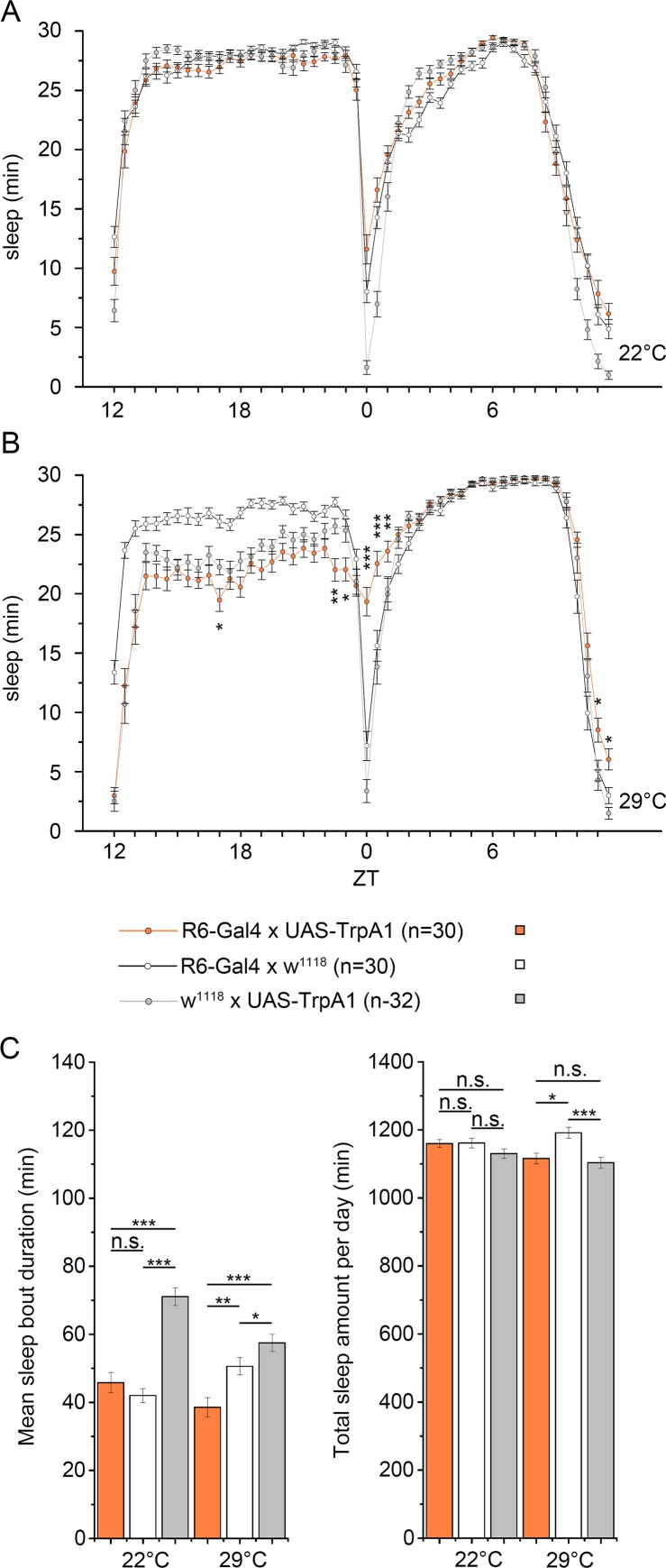
Activation of the PDF-expressing sLNvs promotes sleep specifically during the time of morning and evening peak activity. (A) At 22°C, *R6>TrpA1* flies showed the same sleep pattern than controls. (B) Activation of the TrpA1 channel at 29°C resulted in increased sleep time specifically during the time of the morning and, to a lesser amount, the evening activity. (C) Mean sleep bout duration but not the total amount of sleep per day was affected by activation of the sLNvs.
