# Supplementary material for: Allatostatin A Signalling in Drosophila Regulates Feeding and Sleep and Is Modulated by PDF
Source: PLoS Genet. 2016 Sep 30;12(9):e1006346. doi: 10.1371/journal.pgen.1006346 (PMC5045179; doi:10.1371/journal.pgen.1006346)
Supplement: S1 Text — (DOC) [file pgen.1006346.s001.doc]

**S1 Text:**

*Recipe for standard Drosophila medium*

5.9 kg corn semolina was mixed with 34 L water, boiled for 3 min, and then constantly and slowly stirred for 4 h while cooling down. The next day, 6 L water, 1.8 kg malt extract, 1.8 kg sugar beet molasses, 0.4 kg soy flour, 0.74 kg yeast powder and 0.25 kg agar-agar were added, and the mixture was boiled for 3 min under constant stirring. When the medium had cooled down to ~ 80°C, 0.1 kg methyl-4-hydroxybenzoate (nipagin) was intermixed.

*Primer sequences used for the amplification of the AstA promoter region*

1.03 kB promoter fragment, AstA^1X^:

sense 5'-GCGCAATTGATGGCTATTTCCCAGCTCCT-3'

antisense 5'-GCCGGATCCAGAGGTTCCGCGGACTAAAT-3'

2.05 kB promoter fragment, AstA^2X^:

sense 5'-GCGCAATTGAGTAGAAGCTGCGCCAGAAG-3'

antisense 5'-GCCGGATCCAGAGGTTCCGCGGACTAAAT-3'

2.74 kB promoter fragment, AstA^3X^:

sense 5'-GCGCAATTGGGGAAAAATCTCCGAAAACC-3'

antisense 5'-GCCGGATCCAGAGGTTCCGCGGACTAAAT-3'
