# Supplementary material for: Allatostatin A Signalling in Drosophila Regulates Feeding and Sleep and Is Modulated by PDF
Source: PLoS Genet. 2016 Sep 30;12(9):e1006346. doi: 10.1371/journal.pgen.1006346 (PMC5045179; doi:10.1371/journal.pgen.1006346)
Supplement: S1 Table — (DOC) [file pgen.1006346.s002.doc]

**S1 Table:** Expression patterns of *AstA^34^-Gal4* and *tsh-Gal80; AstA^34^-Gal4*.

Occasionally, we noticed several additional somata with weak AstA IR in the CNS. Since these cells were not included in the *AstA^34^-Gal4* expression pattern and projections were not labelled, we did not analyse them further.

| Organ | GFP expression  AstA^34^-Gal4 | GFP expression *tsh-Gal80; AstA^34^-Gal4* | AstA immunoreactivity^*^ | GPF or LacZ  expression *AstA^1^-Gal4 ^#^* |
| --- | --- | --- | --- | --- |
| central brain | 2-3 per hemisphere in the posterior lateral protocerebrum | + | +  (PLP cells) | + |
|  | 2–4 per hemisphere in the lateral cell body rind (LCBR cells) | + | - | - |
| optic lobes | small number of cells in the medulla | + | + | + |
| TAG | 3 pairs of abdominal cells at the posterior end of the TAG (innervate the gut) | - | + (DLAa^§^ cells in abdominal neuromeres) | + |
| peripheral NS | 2 pairs of cells on segmental nerves exiting the thoracic part of the TAG | - | + (peripheral cells on the wing and haltere nerves) | + |
| gut | EECs in the posterior midgut | + | + | + |

*AstA IR detected in this study, as well as from Yoon and Stay (1995) and Santos et al. (2007), which are consistent with our findings. For nomenclature, see Yoon and Stay (1995).

#Hergarden et al. (2012)

^§^The designation DLAa (dorsolateral abdominal a) neurons seems not fully accurate because part of their somata lie centrally or slightly ventrally within the adult thoracico-abdominal ganglion (TAG).

DLAa dorsolateral abdominal a, EECs enteroendocrine cells, LCBR lateral cell body rind, NS nervous system, PLP posterior lateral protocerebrum, TAG thoracico-abdominal ganglion, VG ventral ganglion, VMA ventromedial abdominal.
