# Supplementary figures and images for: Allatostatin A Signalling in Drosophila Regulates Feeding and Sleep and Is Modulated by PDF
Source: PLoS Genet. 2016 Sep 30;12(9):e1006346. doi: 10.1371/journal.pgen.1006346 (PMC5045179; doi:10.1371/journal.pgen.1006346)

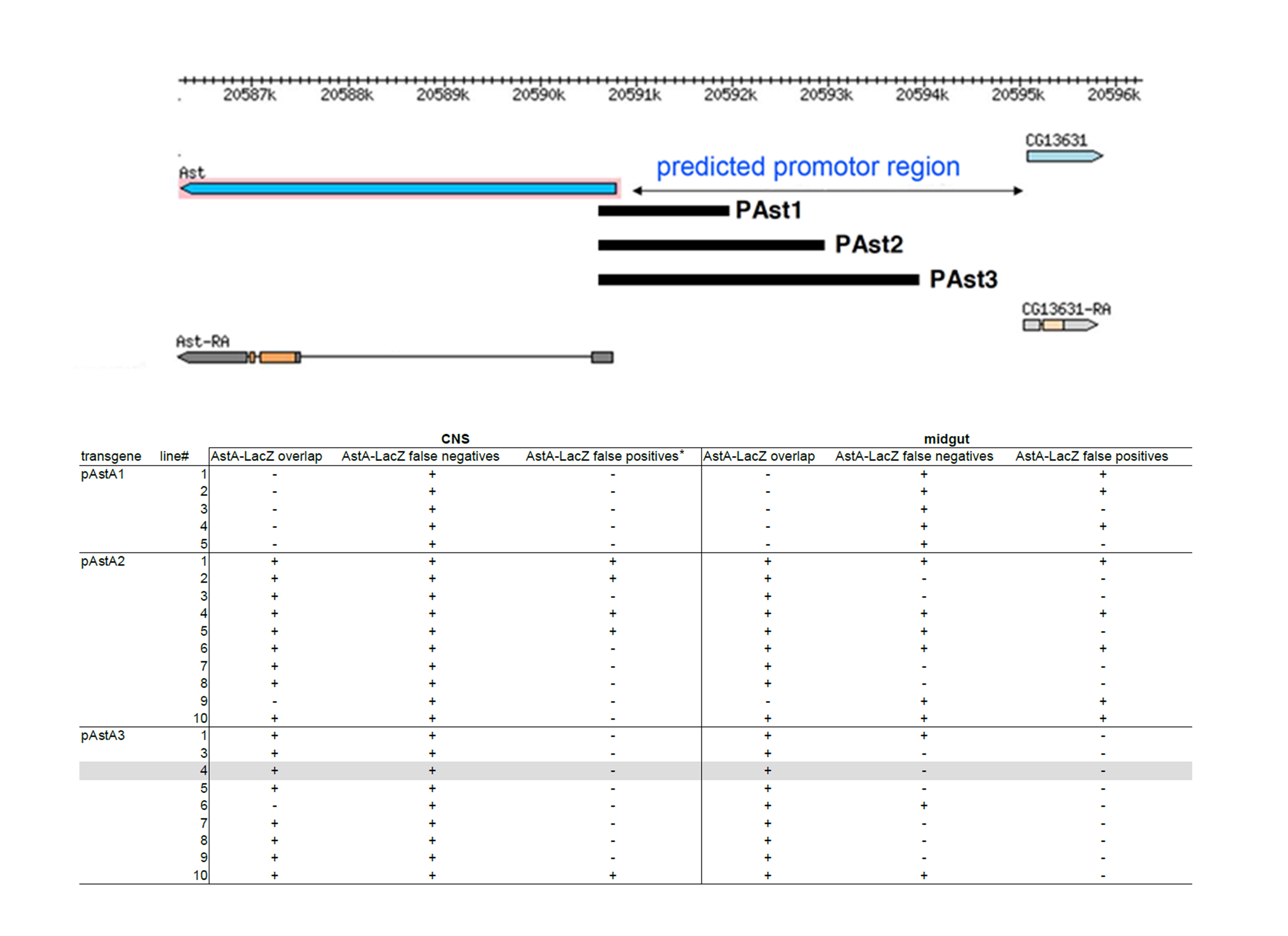

Supplement: S1 Fig — AstA-LacZ overlap: Gal4-UAS-LacZ expressing cells are AstA-immunpositive, AstA-LacZ false positives: Gal4-UAS-LacZ expressing cells are not AstA-immunopositive, AstA-LacZ false negatives: AstA-immunopositive cells not contained in the Gal4-UAS-LacZ expression pattern. The following primer sets were used to amplify the respective promoter regions: pAstA1: 5’-GCGCAATTGATGGCTATTTCCCAGCTCCT-3’ 5’-GCCGGATCCAGAGGTTCCGCGGACTAAAT-3’ pAstA2: 5’-GCGCAATTGAGTAGAAGCTGCGCCAGAAG-3’ 5’-GCCGGATCCAGAGGTTCCGCGGACTAAAT-3’ pAstA3: 5’-GCGCAATTGGGGAAAAATCTCCGAAAACC-3’ 5’-GCCGGATCCAGAGGTTCCGCGGACTAAAT-3’ (TIF) [file pgen.1006346.s003.tif]

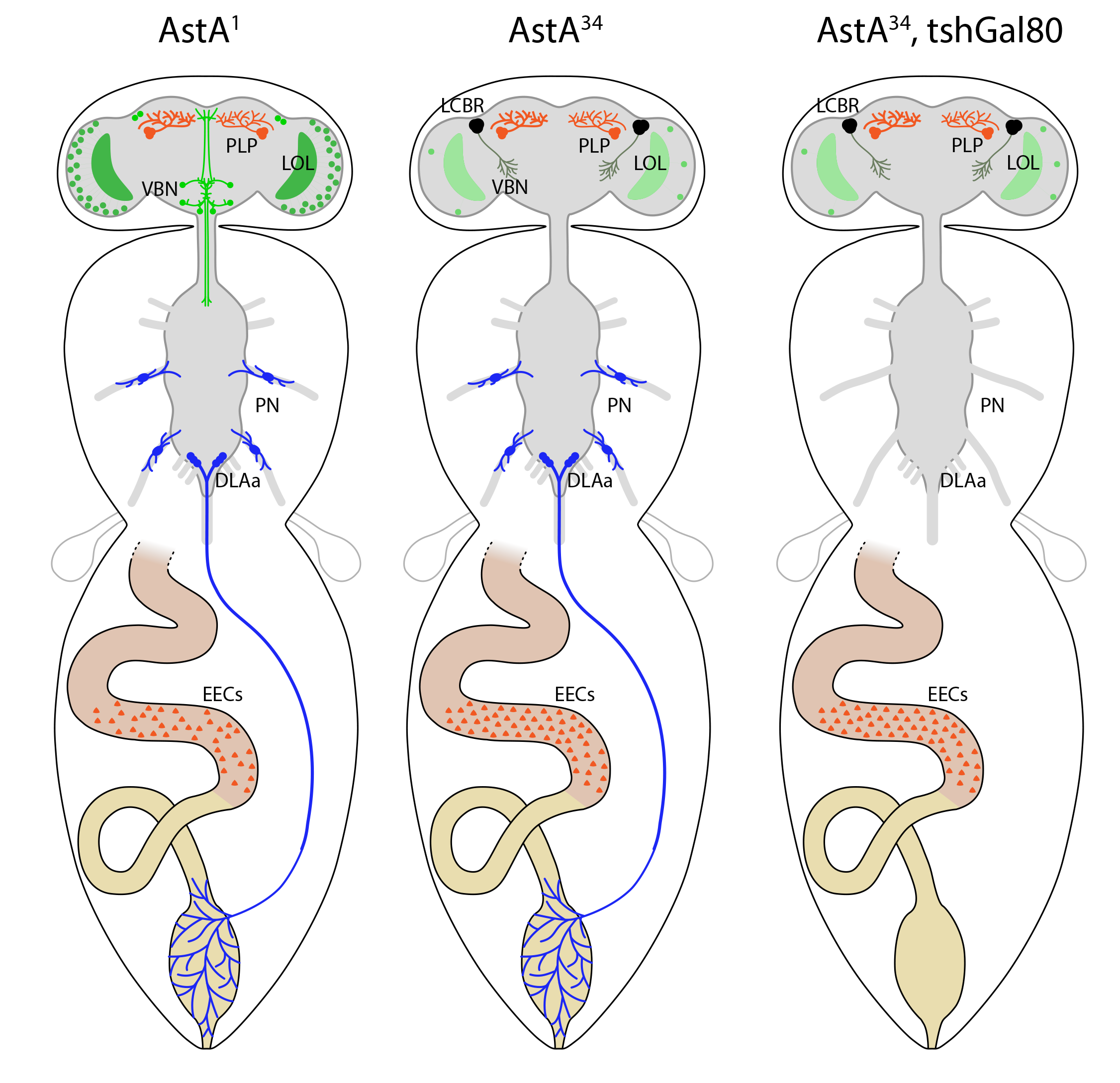

Supplement: S2 Fig — AstA neurons in the posterior lateral protocerebrum (PLP cells) are in red, the dorsolateral abdominal AstA neurons (DLAa) in the thoracico-abdominal ganglion and the peripheral neurons (PN) are in blue, other AstA neurons in the ventral brain (VBN) and lateral optic lobes (LOL) are in green. AstA-expressing enteroendocrine cells (EECs) in the posterior midgut are represented by red triangles. (TIF) [file pgen.1006346.s004.tif]

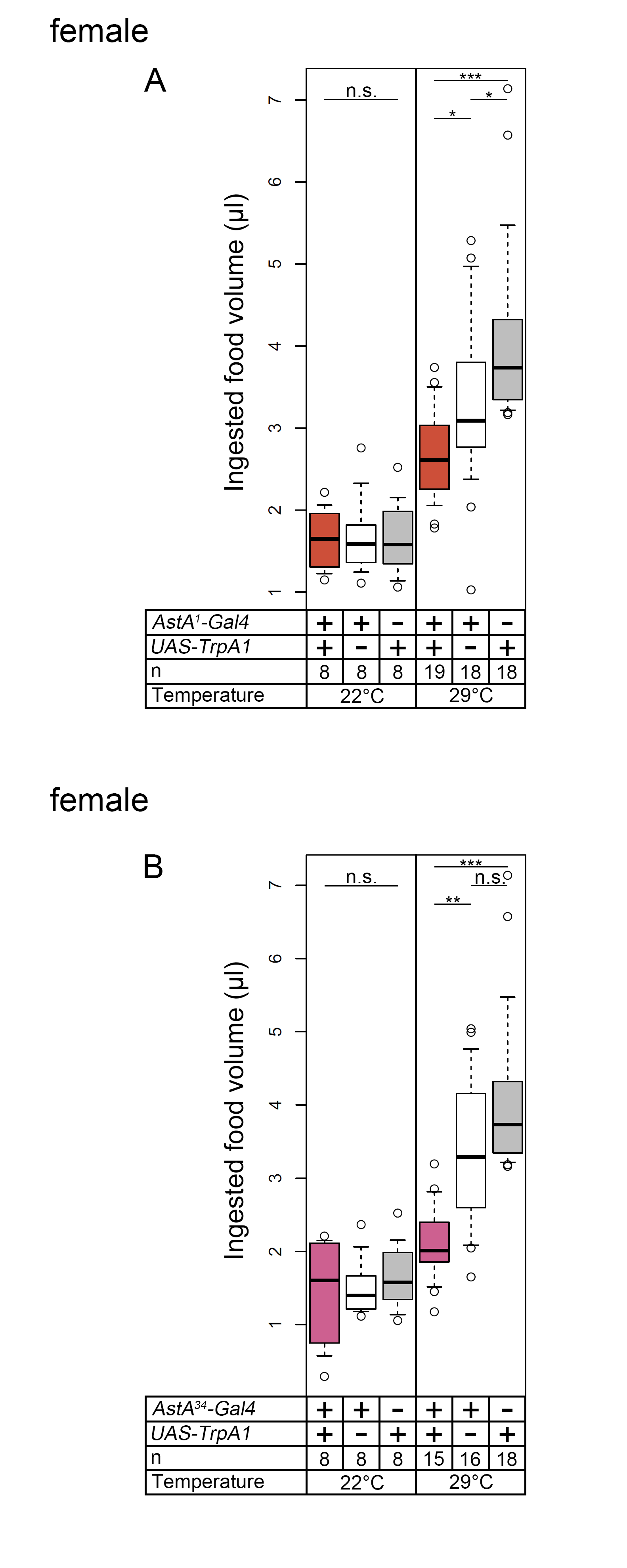

Supplement: S3 Fig — (A) AstA1>TrpA1. (B) AstA34>TrpA1 and respective controls. * p ≤ 0.05, ** p ≤ 0.01, *** p ≤ 0.001. (TIF) [file pgen.1006346.s005.tif]

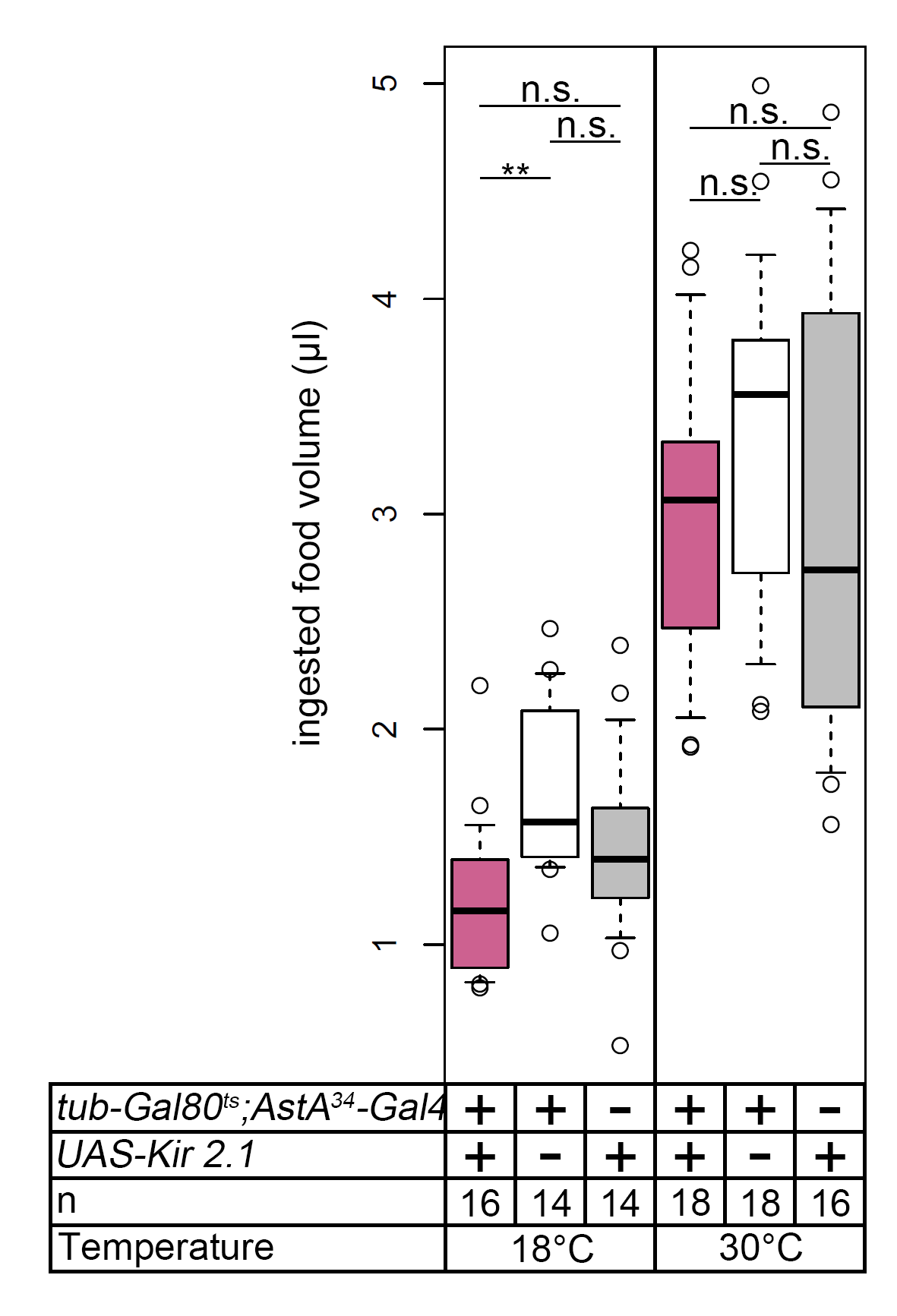

Supplement: S4 Fig — (TIF) [file pgen.1006346.s006.tif]

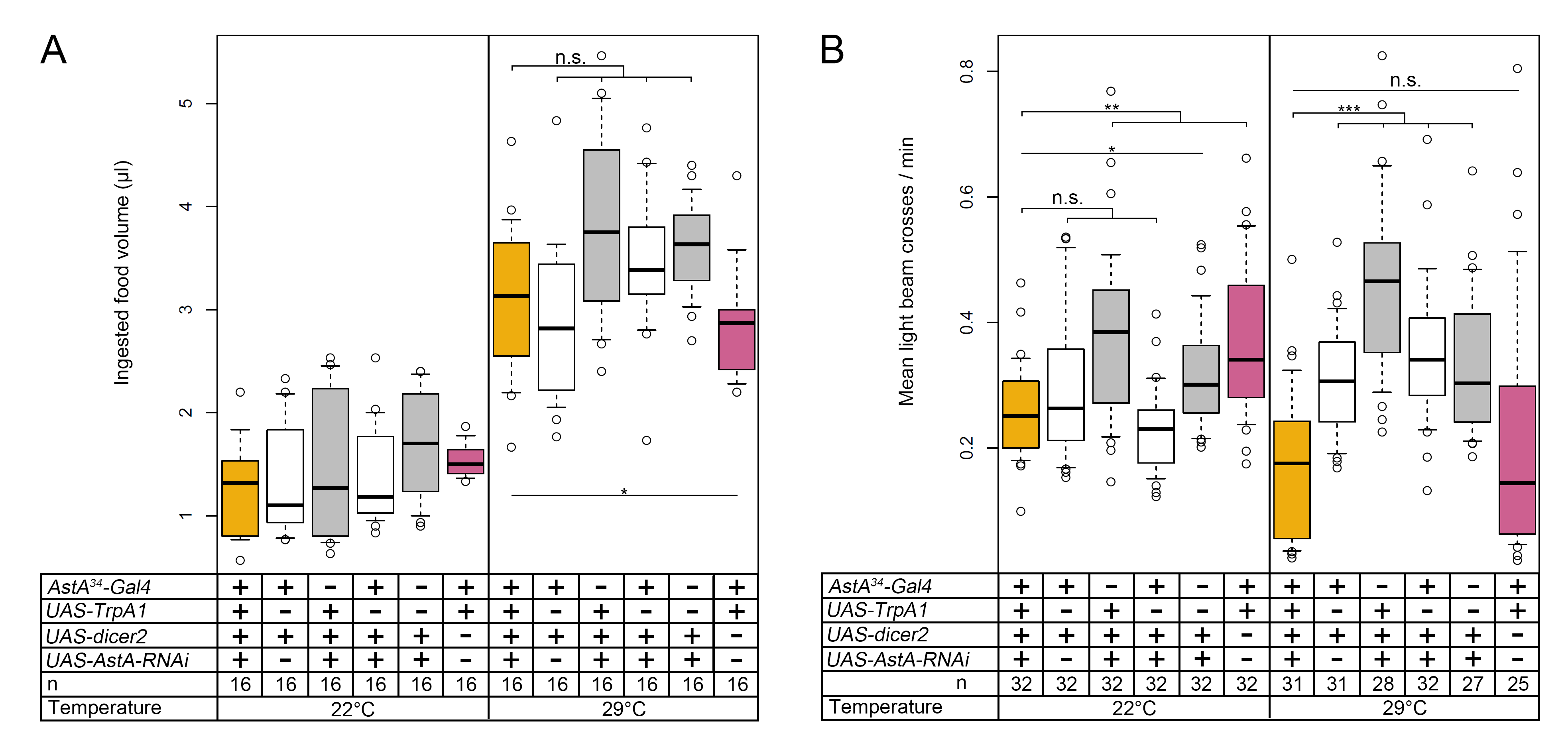

Supplement: S5 Fig — At 22°C, food intake (A) and locomotor activity (B) of AstA34>TrpA1 flies did not show significant differences to controls. At 29°C, thermogenetic activation of AstA34 cells resulted in lower food intake (A) and locomotor activity (B). AstA34>TrpA1/UAS-dcr-2; AstA-RNAi flies were not significantly different in food consumption to AstA34>TrpA1, but showed a significantly reduced locomotor activity. * p ≤ 0.05. ** p ≤ 0.01, *** p ≤ 0.001. (TIF) [file pgen.1006346.s007.tif]

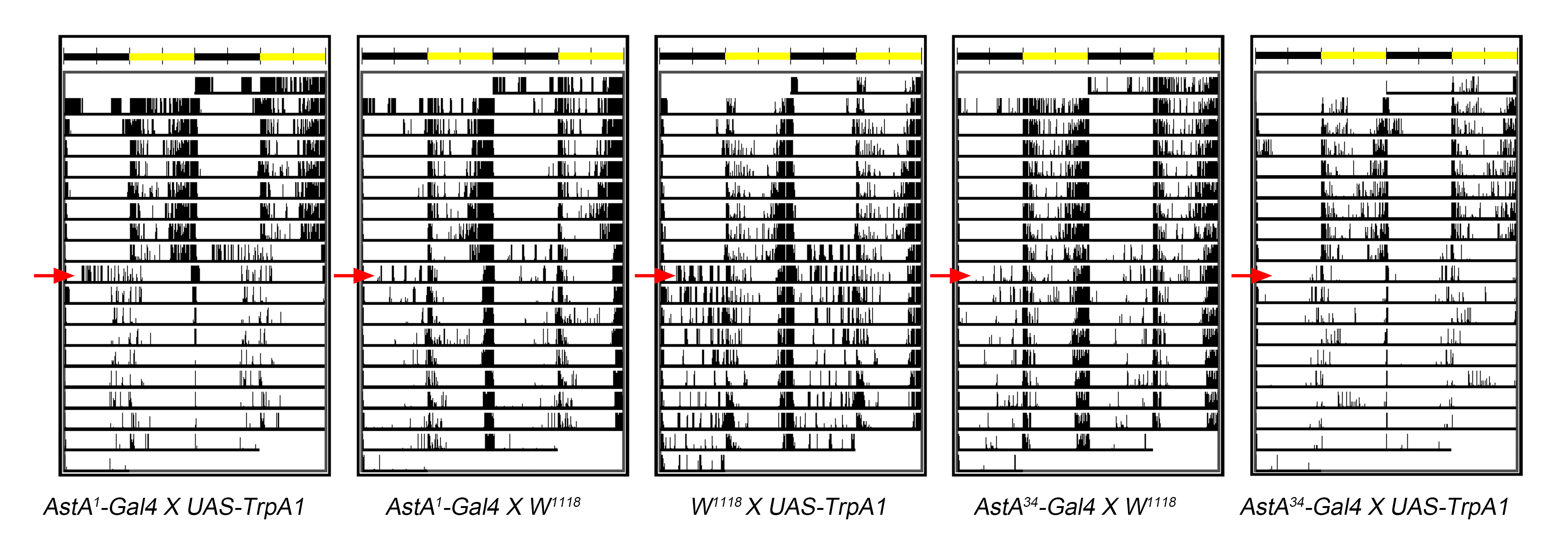

Supplement: S6 Fig — Examples of double-plotted single fly actogramms underlying the results shown in Fig 4. Flies were initially kept at 22°C, then temperature was raised to 29°C at the time point indicated by a red arrow. (TIF) [file pgen.1006346.s008.tif]

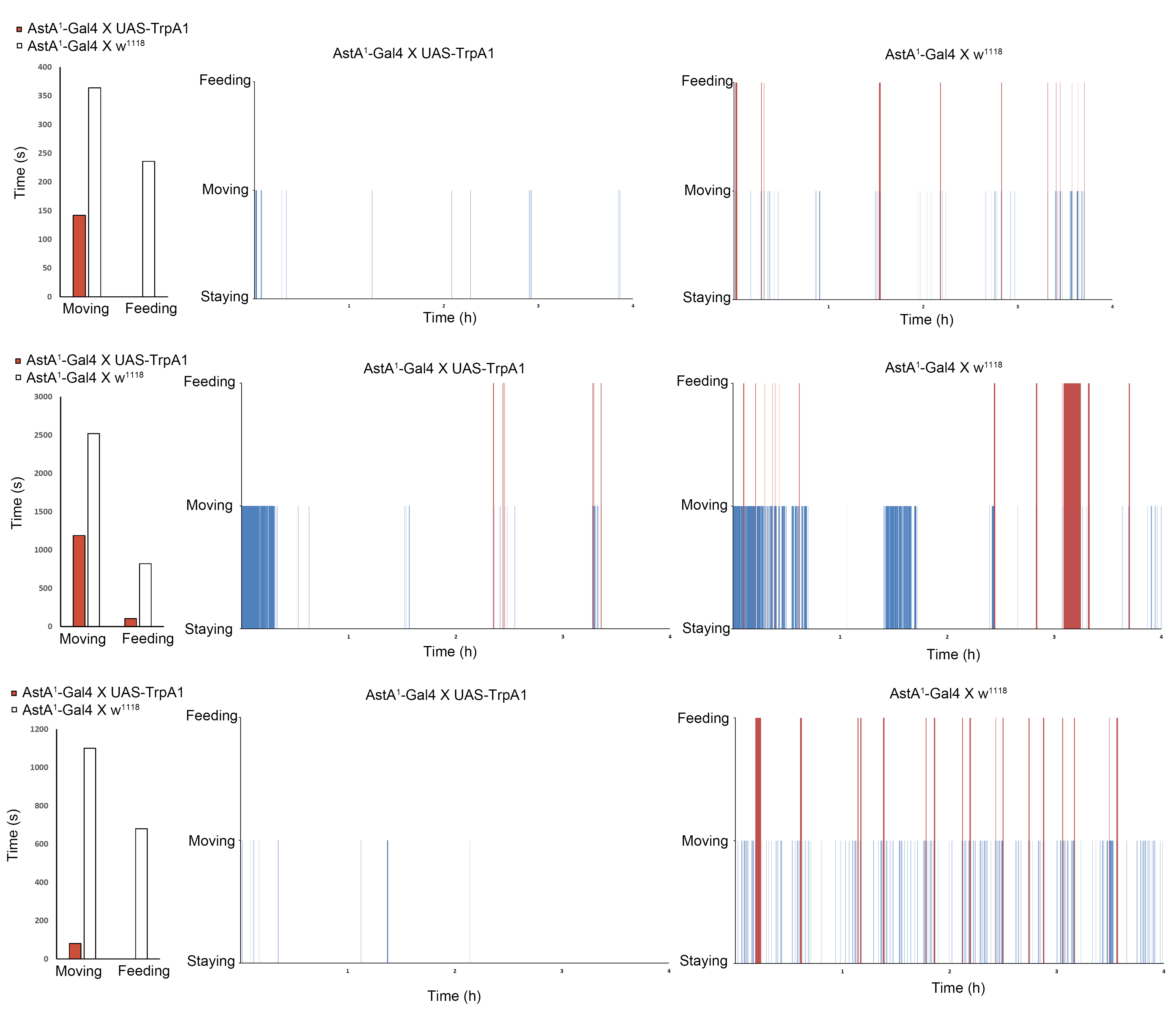

Supplement: S7 Fig — Individual flies were filmed for 4 hours in a modified CAFE assay (three flies per genotype). Behaviour was categorized as "not moving", "moving" and "feeding". AstA1>TrpA1 flies with activated AstA1 cells moved and consumed less than controls. (TIF) [file pgen.1006346.s009.tif]

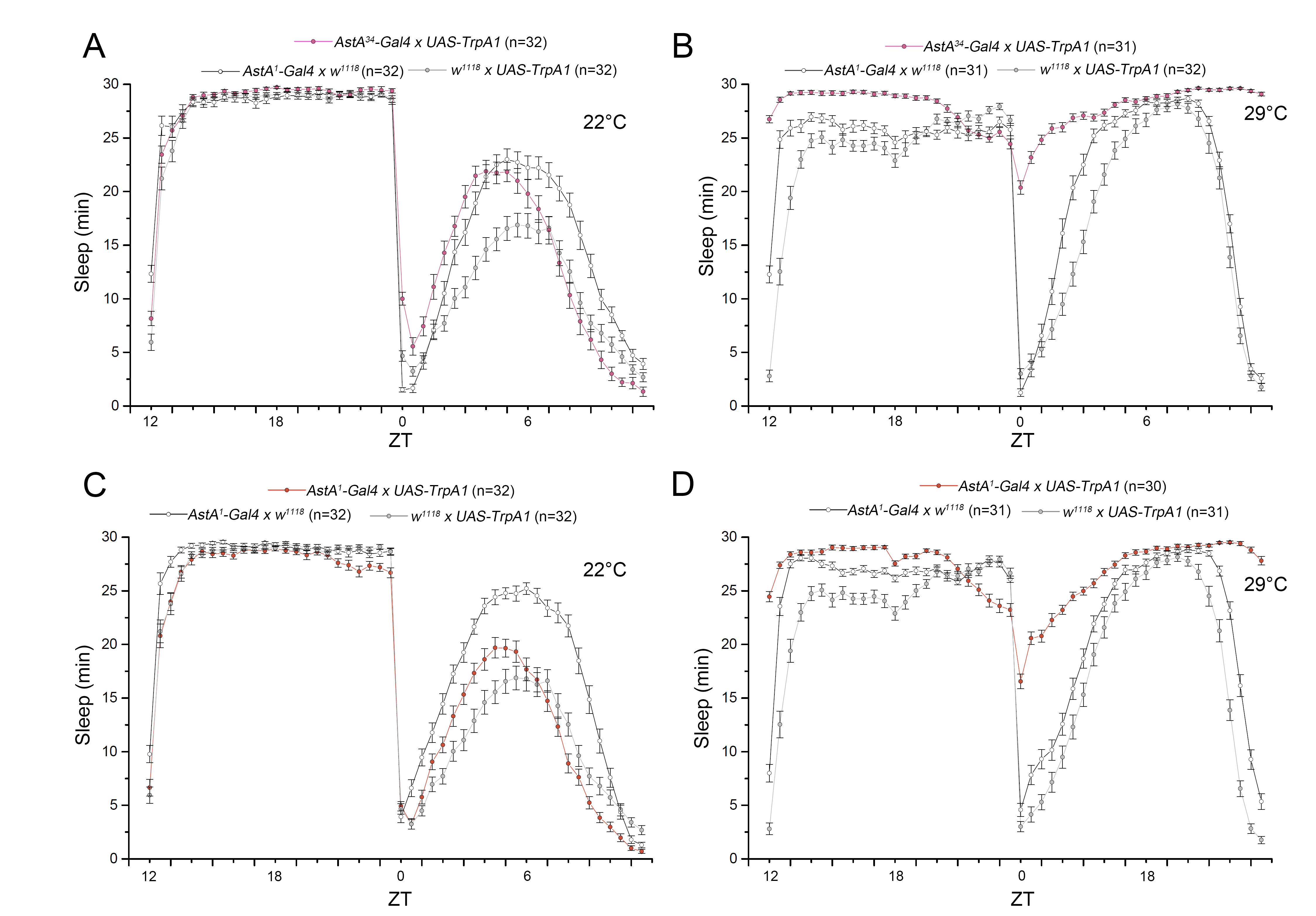

Supplement: S8 Fig — At 20°C, AstA34>TrpA1 (top left) and AstA1>TrpA1 females (bottom left) did not sleep more than controls. Activation of the TrpA1 channel by 29°C resulted in increased sleep time of AstA34>TrpA1 (top right) and AstA1>TrpA1 (bottom right) females during the light phase from ZT0 to ZT12. (TIF) [file pgen.1006346.s010.tif]

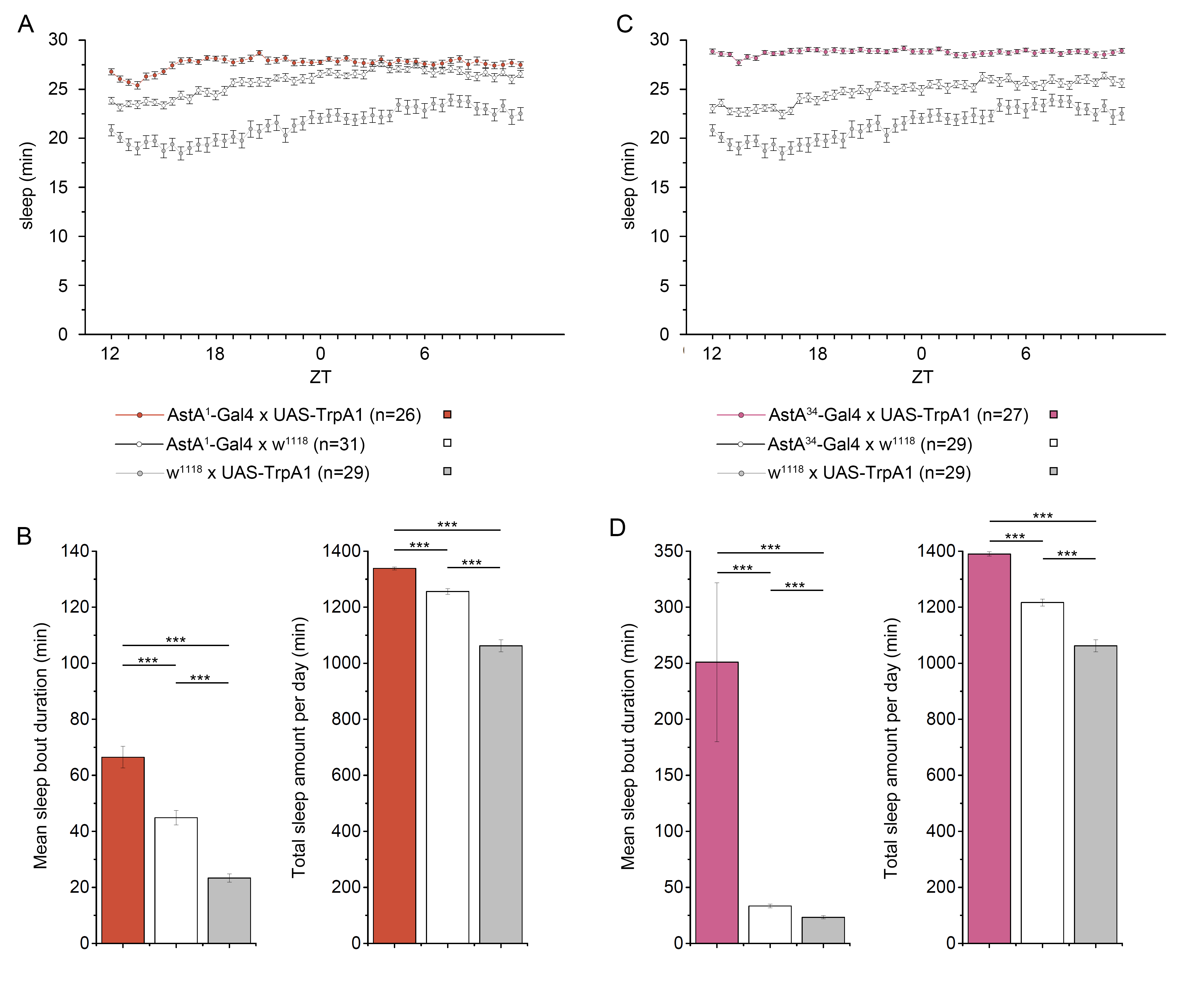

Supplement: S9 Fig — (TIF) [file pgen.1006346.s011.tif]

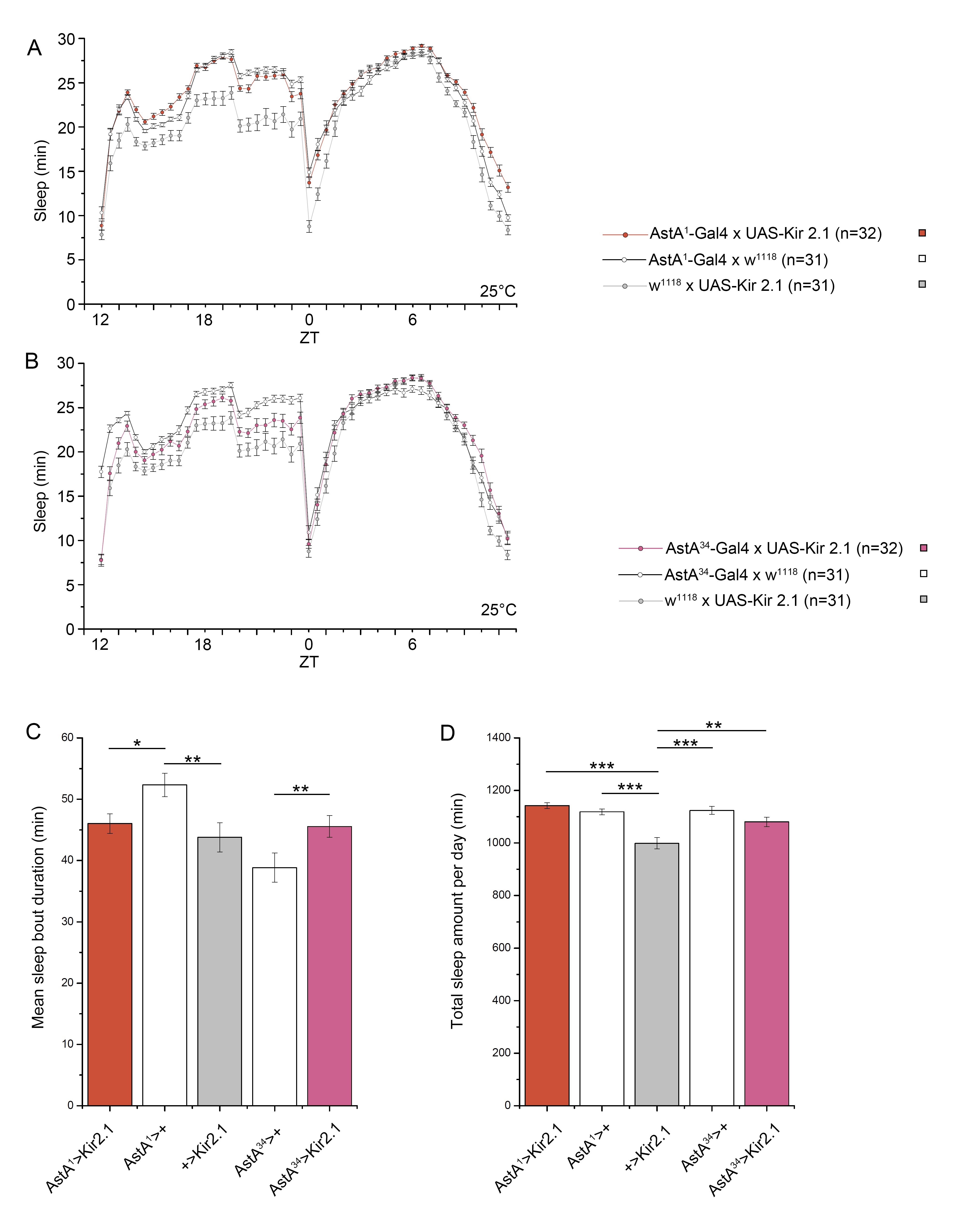

Supplement: S10 Fig — Constitutive silencing of AstA cells by ectopic expression of Kir2.1 did not alter sleep behaviour in AstA34>Kir2.1 (A) and AstA1>Kir2.1 (B) flies. The mean sleep bout duration (C) and the total amount of sleep per day (D) of AstA34>Kir2.1 and AstA1>Kir2.1 is not significantly different to all controls. (TIF) [file pgen.1006346.s012.tif]

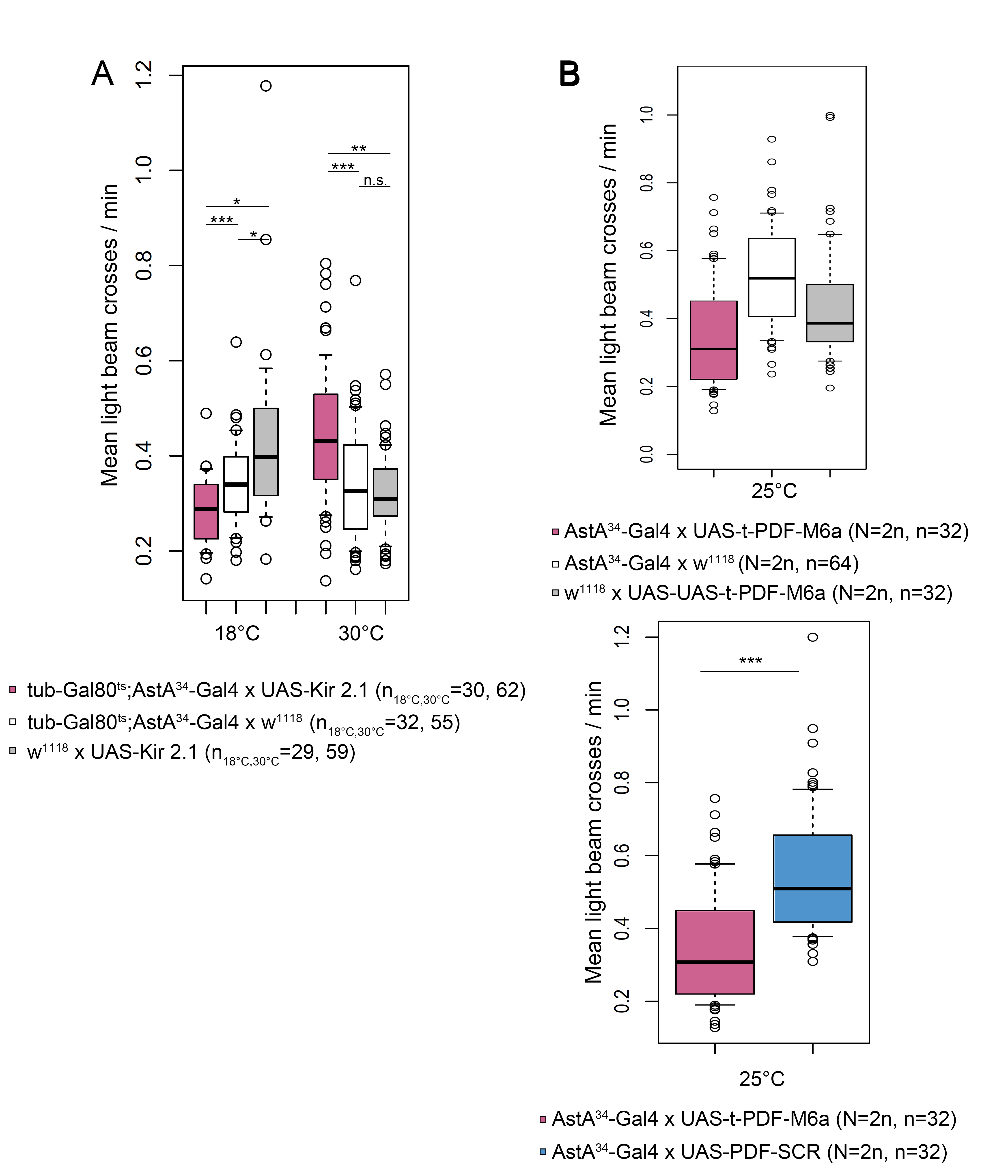

Supplement: S11 Fig — A) Conditional silencing of AstA34 cells increases the mean locomotor activity. B) Ectopic expression of t-PDF in AstA34 cells decreases the mean locomotor activity. (TIF) [file pgen.1006346.s013.tif]

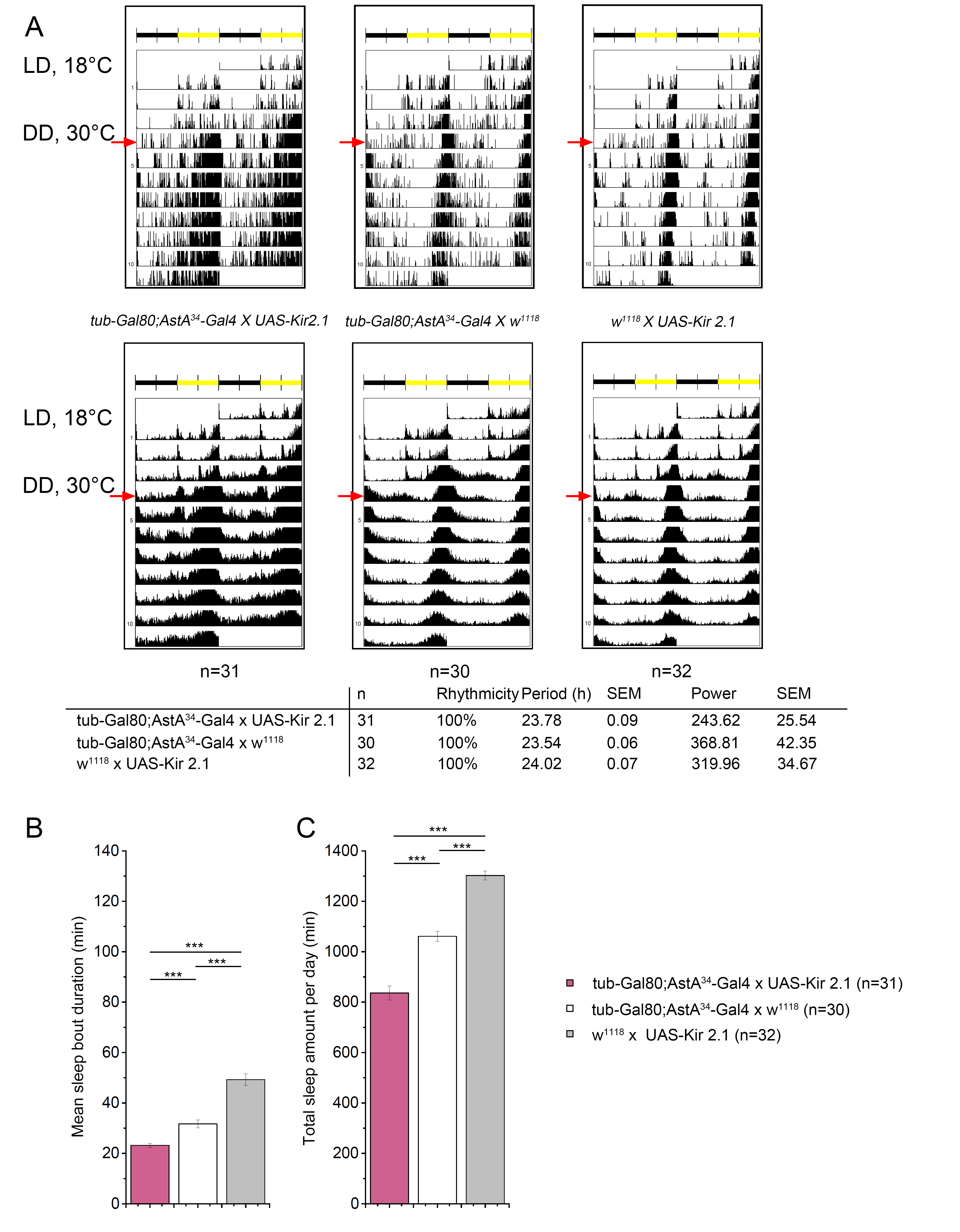

Supplement: S12 Fig — (A) Actogramms of single flies show that locomotor activity increases during the subjective day and night upon silencing of AstA34 cells. Rhythmicity and period is not affected. Both the duration of sleep bouts (B) and total sleep (C) is reduced. (TIF) [file pgen.1006346.s014.tif]

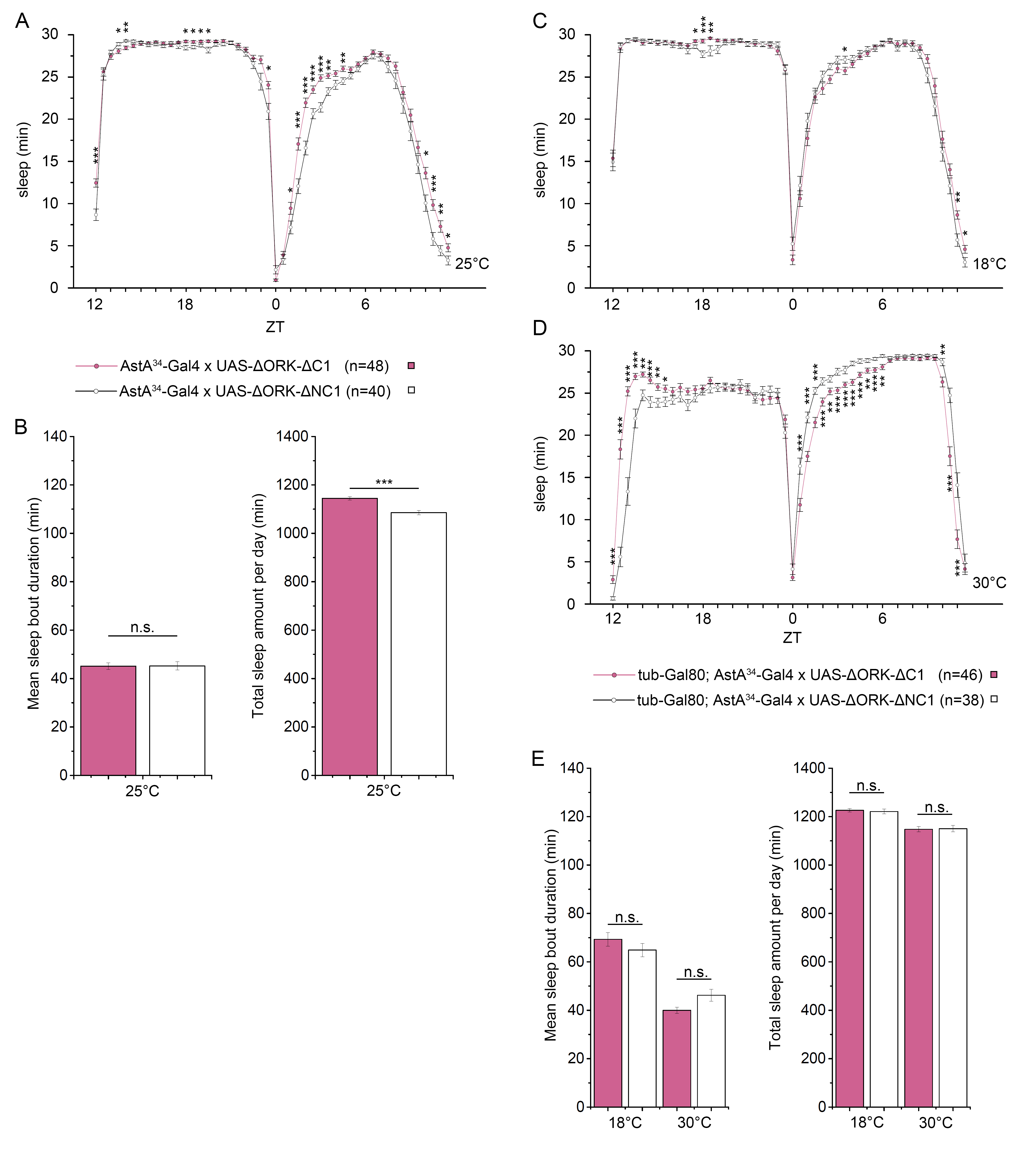

Supplement: S13 Fig — The effect on sleep of constitutive (A-B) and conditional (C-E) silencing of AstA34 cells by UAS-ΔORK under LD12:12 (A-B) Constitutive silencing let to a slight increase in the total amount of sleep (B), mostly due to increased sleep during the day (A). This effect is opposite of the expected decrease upon AstA cell silencing. (C-E) Conditional silencing did not affect total sleep or sleep bout duration (E), yet sleep is increased during the end of the evening activity and decreased during the first half of the photophase. (TIF) [file pgen.1006346.s015.tif]

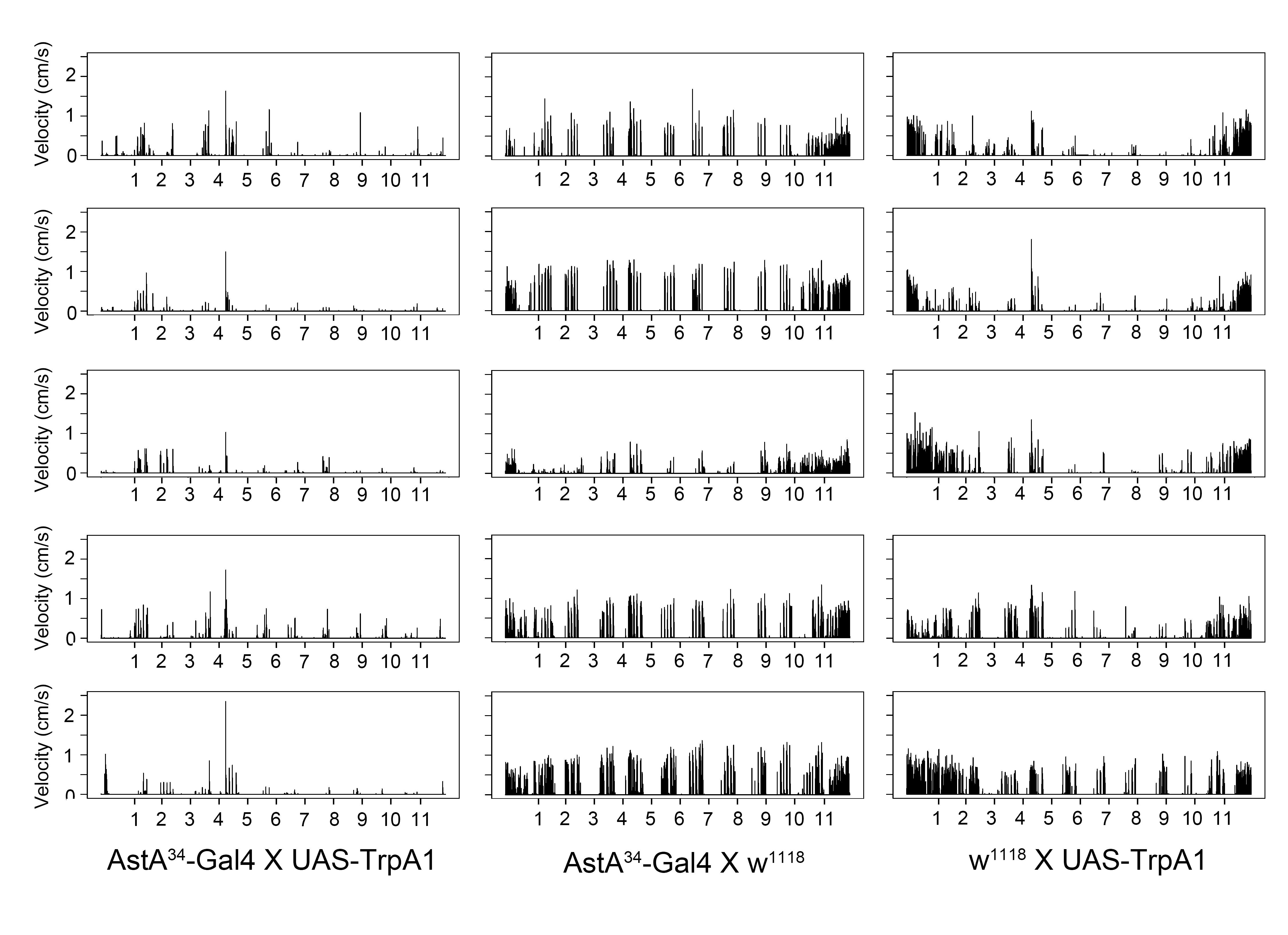

Supplement: S14 Fig — While AstA34>TrpA1 flies walked less (leading to a reduced average velocity), the maximum speeds when moving where not different to control flies, suggesting that the reduced locomotor activity is not due to motor impairment. (TIF) [file pgen.1006346.s016.tif]

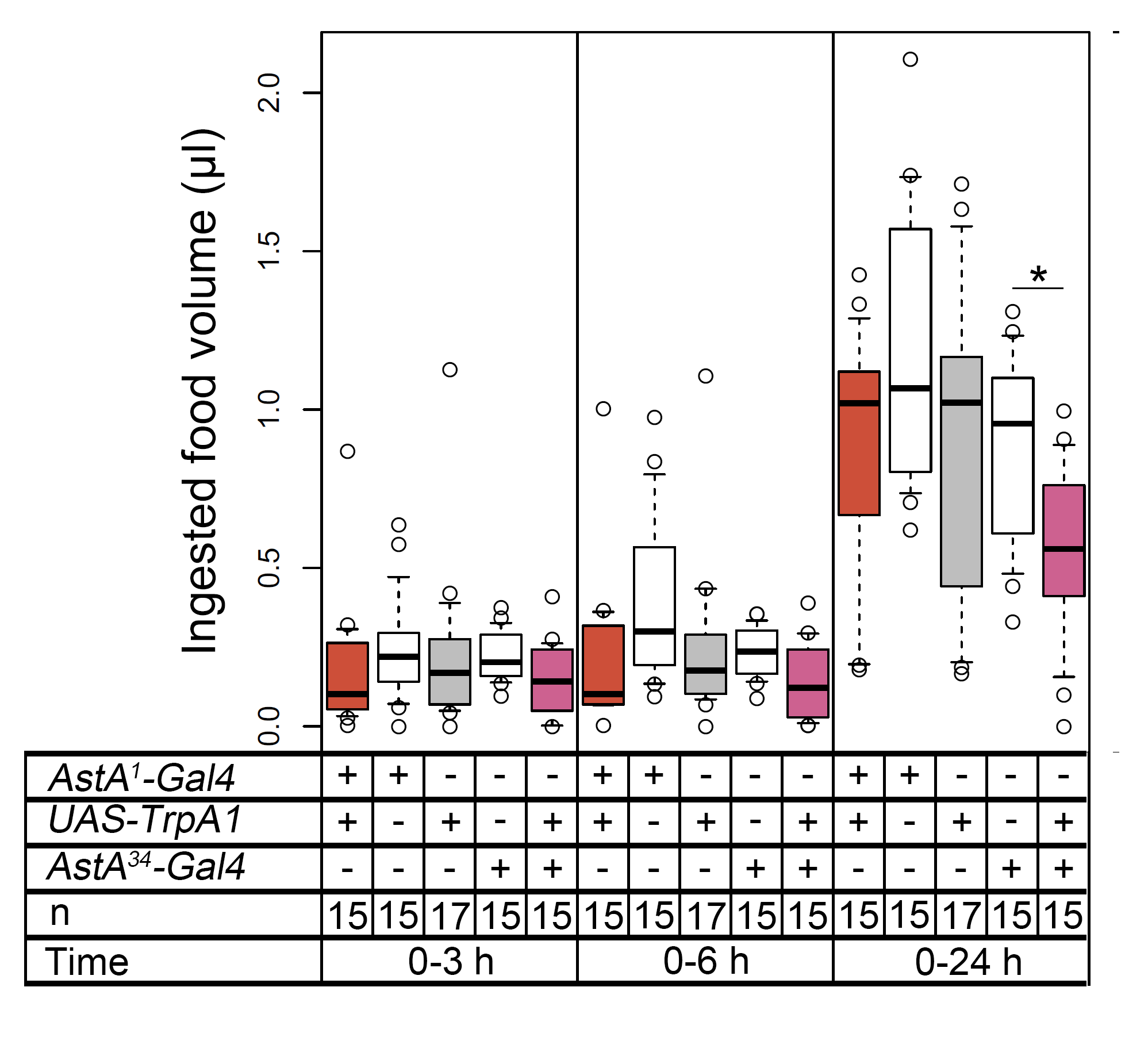

Supplement: S15 Fig — AstA34>TrpA1 and AstA1>TrpA1 flies were kept for 1 day at 22°C, then 2 days at 29°C in the CAFE assay. Afterwards, flies were put back again to 22°C, and food consumption was summed up for the first 3, 6 and 24h. (TIF) [file pgen.1006346.s017.tif]

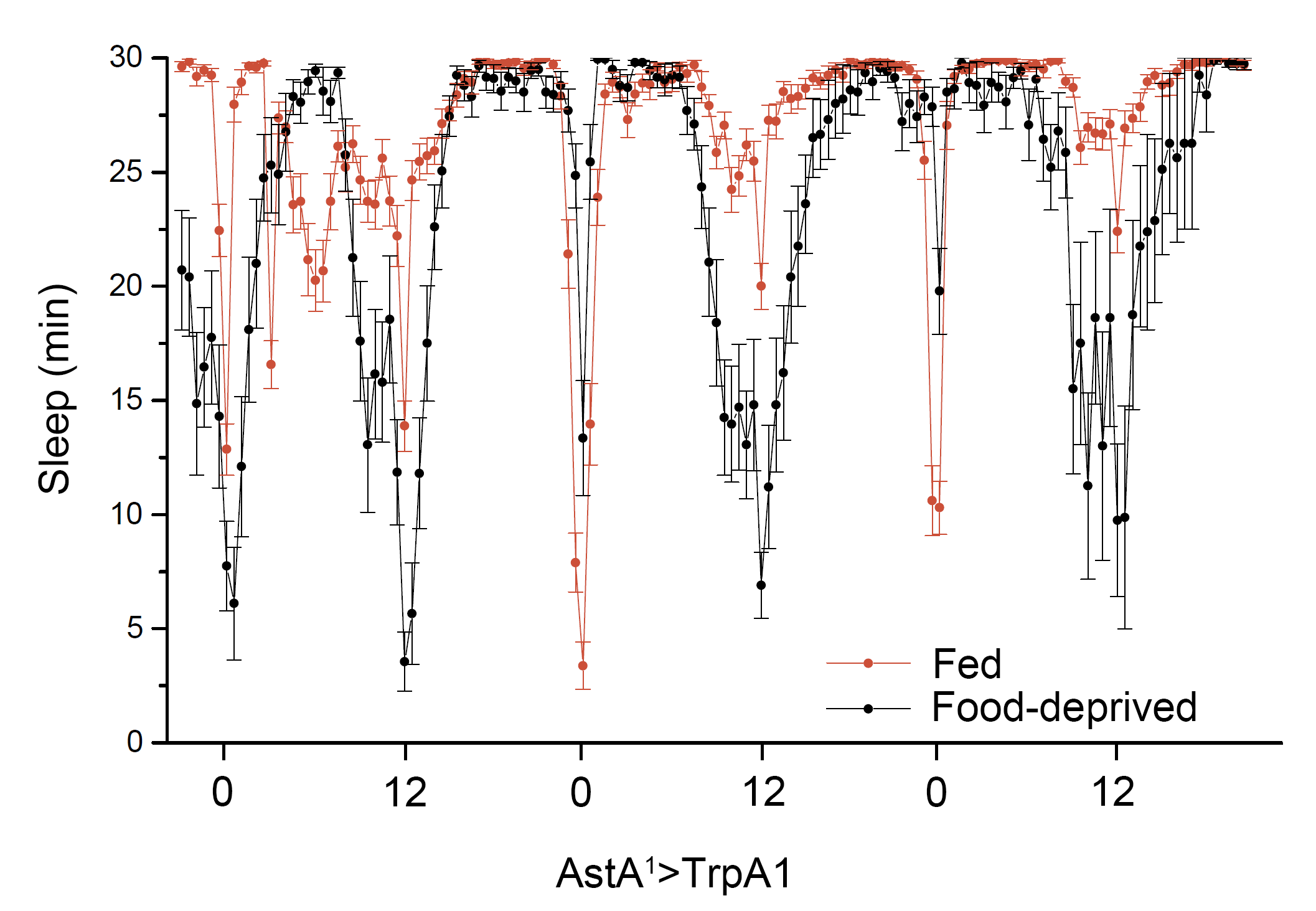

Supplement: S16 Fig — Flies were kept at 20°C in LD12:12 on normal food, and then transferred to DAM glass tubes and switched to 29°C and feeding/starvation-conditions at ZT8 at the start of locomotor activity monitoring (n = 32). (TIF) [file pgen.1006346.s018.tif]

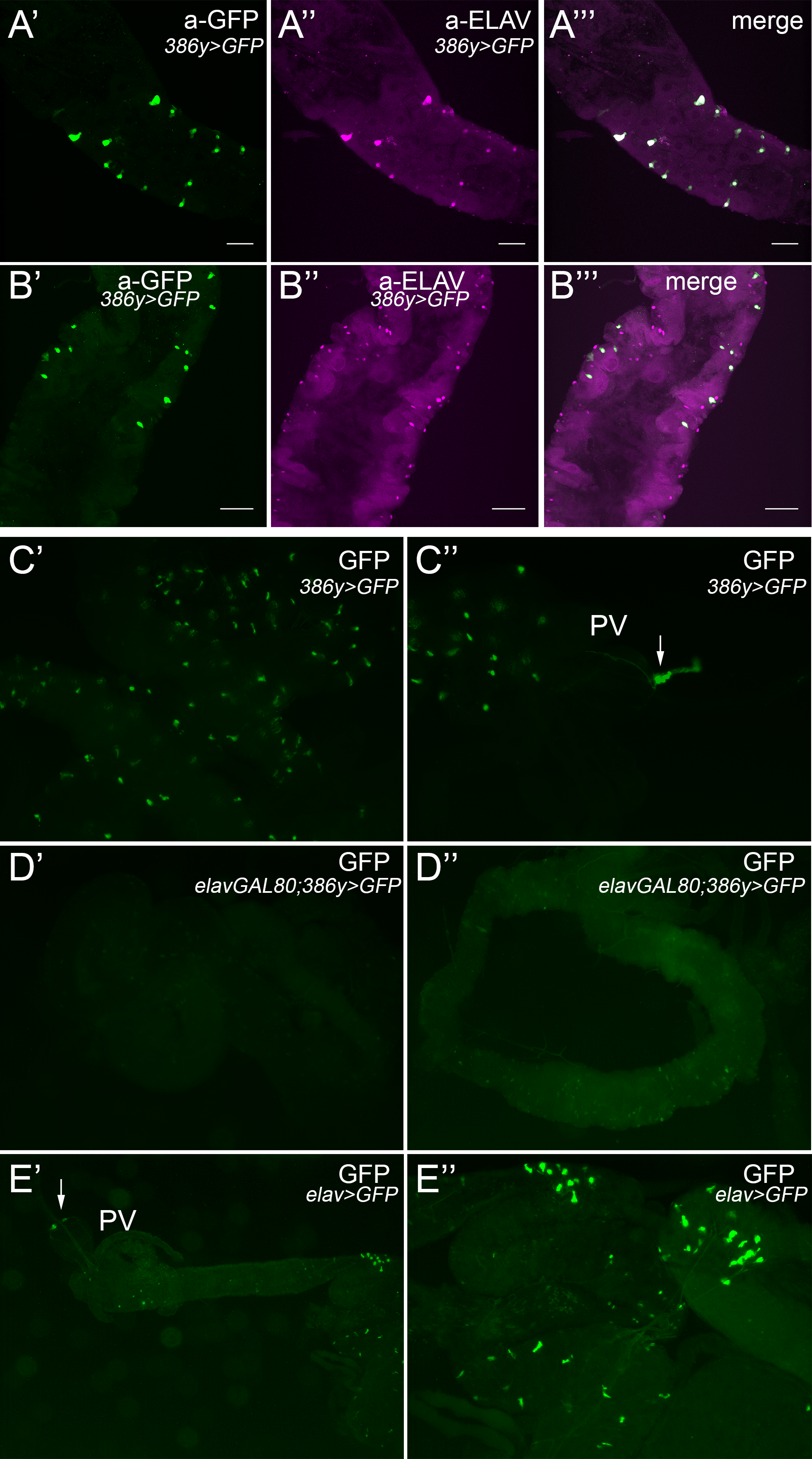

Supplement: S17 Fig — A and B): GFP expression (anti-GFP staining, green in A'/B') driven by the peptidergic cell marker 386y-Gal4 (Taghert et al. 2001, Reiher et al. 2011) colocalises with a-ELAV immunoreactivity (magenta, A''/B'') as seen in the merged pictures A'''/B'''. Scale bar = 50 μm. C and D): 386y-Gal4 driven GFP expression (C) is suppressed by co-expression of elav-GAL80 (D). Widefield pictures taken with a CCD camera with an exposure time of 3.75 s (C'), 2.5 s (C''), 5 s (D') and 7.5 s (D'). All other camera settings were kept constant. E): elav-Gal4-driven native GFP expression in EECs. Arrows point to neurons in the proventricular ganglion. (TIF) [file pgen.1006346.s019.tif]

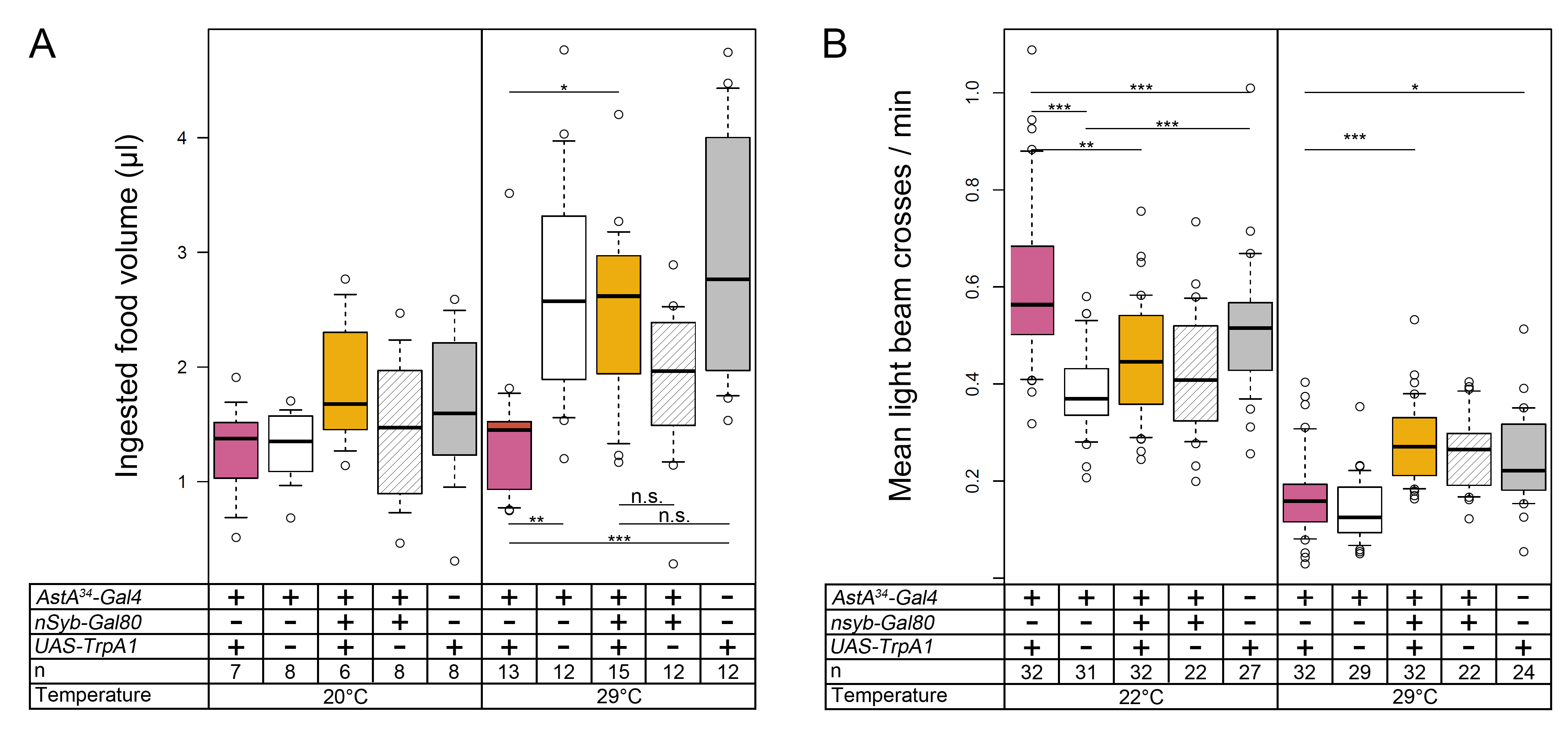

Supplement: S18 Fig — Thermogenetic activation of AstA34 cells resulted in significant lower food consumption (A) and locomotor activity (B) compared to controls. Food intake (A) and locomotor activity (B) of nsyb-Gal80; AstA34>TrpA1 were not significantly different to controls, but significantly higher than AstA34>TrpA1 at 29°C. * p ≤ 0.05. ** p ≤ 0.01, *** p ≤ 0.001. (TIF) [file pgen.1006346.s020.tif]
